# Path dependency and the rescuing of the biomedical research enterprise

**DOI:** 10.3389/fmedt.2025.1683835

**Published:** 2026-01-12

**Authors:** Zaher Nahle

**Affiliations:** 1 Center for a Humane Economy, Bethesda, MD, United States; 2 Ivyctory Solutions, Los Angeles, CA, United States

**Keywords:** animal testing, CDC, drug development, EPA, FDA, FDA modernization Act 2.0 and 3.0, NIH, new alternative methods (NAMs)

## Abstract

In 2025, three U.S. agencies within the Department of Health and Human Services (FDA, NIH, CDC) alongside EPA and the Departments of the Navy and Veterans Affairs, began substituting animal testing applications with reliable, human-relevant methods. The impact of this shift in public policy taking place at agencies historically bullish on animal testing is still reverberating in the United States and around the world. Here, we examine the circumstances that enabled such momentous reforms, including the role of the FDA Modernization Act 2.0 in advancing new alternative methods, collectively referred to as NAMs. We explain how animal testing, despite its poor value in predicting the safety and efficacy of drugs in humans, came to dominate drug discovery, basic sciences, and environmental toxicity assessments since the inception of the Federal Food, Drug, and Cosmetic Act (Federal FD&C Act) in 1938. Specifically, we identify critical junctures, including catastrophic government decisions, that made the overall research enterprise acutely dependent on animals, leading to the existing predicament—an indefensible 92% failure rate in translating drugs from preclinical studies to actual therapies. Notably, our analysis chronicles events through the lens of “path dependency,” a social sciences phenomenon that occurs when faulty past decisions lock-in future action. Finally, we recognize that our narrative is a departure from the medical establishment account or the talking points of powerful interest groups, including some in the academic elite who continue to shore up animal-centric paradigms in drug development for reasons we also outline.

## 1 Introduction

This review presents a no-frills account of the rise and decline of animal testing in preclinical studies starting with the enactment of the Federal FD&C Act of 1938 and ending with the pivot towards animal-free science in 2025. It focuses on path dependency, public policy and NAMs affairs in the context of the United States. That said, the work is portable to other settings, including those at the Organization for Economic Co-operation and Development (OECD) countries and the European Union (E.U.), where vibrant research and development activities using NAMs are taking place and where many regulatory decisions are modeled after regulations initiated by U.S. agency counterparts.

Of note, while this work was under review, a strong commitment to animal testing alternatives by the U.K. government was announced (1). It surpassed—in relative size and specificity—national roadmaps declared elsewhere, including in the U.S. As detailed in the U.K. plans on November 11, the use of dogs and non-human primates (NHPs) in medicines testing will be cut by at least 35% by 2030. That is in addition to adopting only DNA-based methods for detecting virus contamination in medicines starting in 2027, replacing botox potency tests on mice with DNA-based methods by 2027 and phasing out skin/eye irritation tests by the end of 2026. A funding commitment of new £75 million (or around $98 million) was allocated to positioning the U.K. as a global leader in this field.

We also make the distinction from the get-go that our overall thesis is predicated on the fundamental belief that publicly funded health studies in particular should advance the knowledge of human-relevant science, not support curiosity-driven research—except when the two overlap. From that standpoint, there are different attitudes in the scientific community today when it comes to animal testing—and that is expected. Ultimately, success of Investigational New Drugs (INDs) or vaccines in drug development is determined by objective and pragmatic tests, namely clinical trials. By contrast, curiosity-driven investigations, including those funded by the National Institutes of Health (NIH), are not required to develop a human relevancy test, and therefore could be inaccurate, or sometimes even misleading, in modeling human diseases.

According to the National Center for Advancing Translational Sciences (NCATS), one of twenty-seven Institutes and Centers at NIH, “*Therapeutic development is a costly, complex and time-consuming process. The average length of time from target discovery to approval of a new drug is about 14 years. The failure rate during this process exceeds 95 percent, and the cost per successful drug can be $2 billion or more*”.

Indeed, the multifactorial drug development process often fails to translate the knowledge from laboratory animals to the clinic because of intrinsic limitations in the models used (predominately animal models). This includes developmental compensation due to germline gene deletion, species-specific differences in immune function and regulatory networks, as well as failure to capture key characteristics like tumor heterogeneity, microenvironmental influences, and the pharmacogenomic diversity of humans. These limitations can systematically inflate efficacy signals, obscure emergent toxicities, and misrepresent resistance pathways, thereby contributing to high late-stage attrition and constraining clinical applicability, despite earlier regulatory approvals.

## 2 Path dependency in discovery research

### 2.1 Animal testing as a prime example of path dependency

When prior decisions dictate future actions by limiting available options to a handful of choices, economists call this phenomenon “path dependency”. This resistance to change can arise when financial interests are at stake and/or when policymakers introduce broad mandates that prove to be shortsighted and not easy to override.

We tolerate instances of path dependency in healthcare, technology and politics quite well, often with little awareness of their constraints or even their existence. For instance, the QWERT sequence on keyboards, designed initially to reduce mechanical jams, is a holdover from the typewriter era. The identical layout was adopted in the digital age, ostensibly not to spook consumers and jeopardize future sales.

But not all cases of path dependency are benign. Animal testing in drug development and scientific research—a textbook example of path dependency—is an ongoing reminder of the generational harm a society endures because of past decisions too stubborn to rectify. Indeed, path dependency associated with the biomedical research enterprise has proven to be among the hardest to overcome. Case in point, despite their poor value in predicting toxicity in humans, animals have been relied on ubiquitously for such purpose at least since 1938, the enactment year of the Federal FD&C Act—a set of U.S. laws that sanctioned animal use in drug development to predict the effects of tested compounds in humans.

### 2.2 Critical junctures that shaped path dependency in drug development

**Figure 1:**
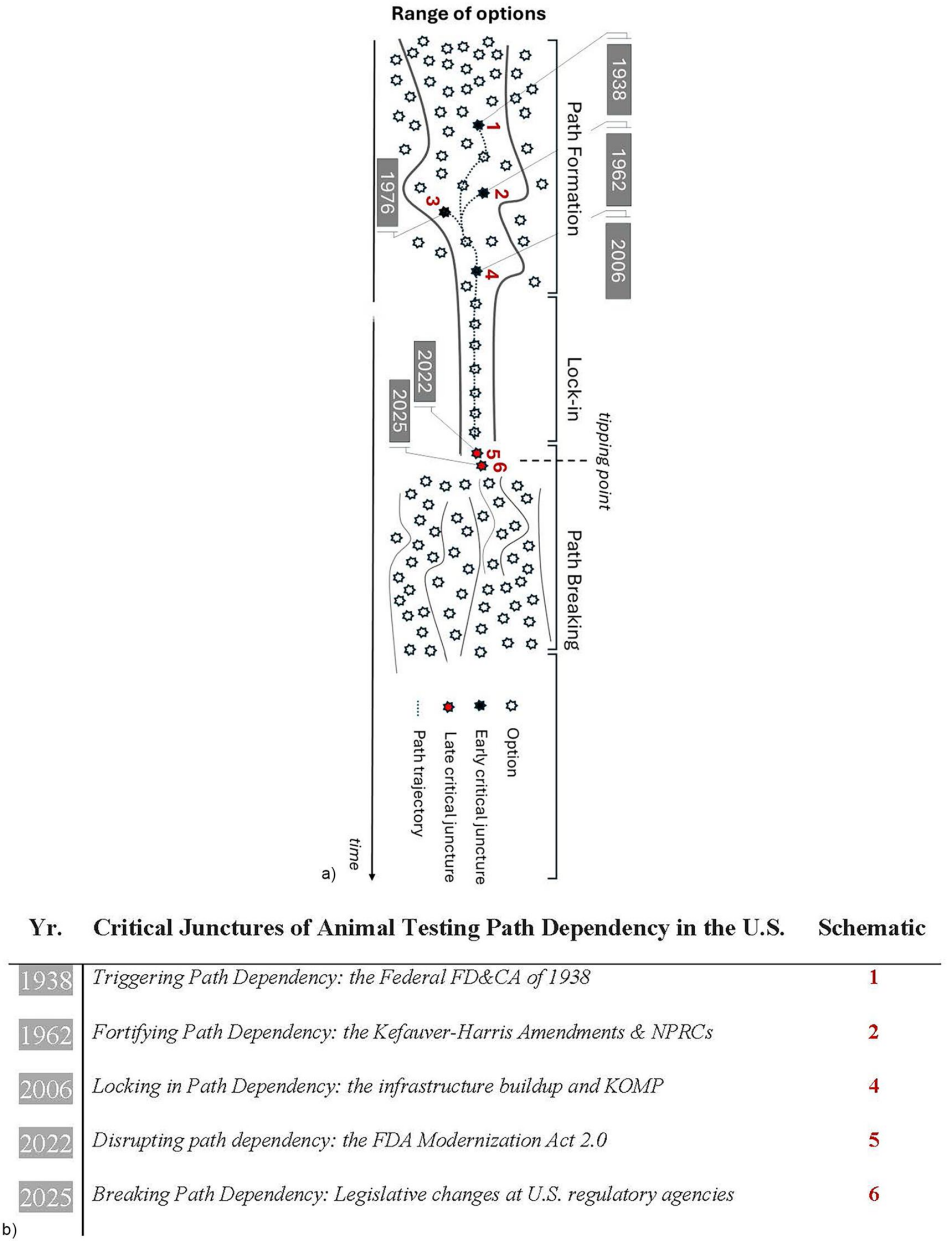
**(a)** visual depiction of path dependency and the critical junctures involved. Three “early” critical junctures (denoted in diagram as 1, 2, and 4) as well as two “late” critical junctures (denoted as 5 and 6) shaped the trajectory of path dependency associated with animal testing. Numbers represent: 1 (the Federal FD&CA era that triggered path dependency in 1938), 2 (fortifying path dependency in 1962 through the Kefauver-Harris Amendments in 1962 and the establishment of the NPRCs), 3 [locking-in path dependency in 2006 through large-scale investments in the animal research enterprises at NIH like the Knockout Mouse Project (KOMP)], 5 (disrupting path dependency in 2022 through the enactment of the FDA Modernization Act 2.0) and 6 (breaking path dependency with the historic pivot at U.S. regulatory agencies in 2025 towards Alternatives). Of note, number 3 represents a key event that caused environmental health assessments to be dominated by animal testing [the Toxic Substances Control Act (TSCA) of 1976] but this aspect is not expanded on in this piece. We elected to include that reference in the diagram to underscore the far reach of animal testing beyond drug development and preclinical research into critical domains like environmental health and chemicals safety testing. **(b)** a tabulated version with brief definitions for clarity.

The defining moments in the genesis of any path dependency that shape it into its rigid, needlelike trajectory (Figure 1) are referred to as “critical junctures”. Such narrowing of options prevents the consideration of better, more efficient alternatives. In our analysis, the rise of animal testing to what became the catch-all test in modeling human health and disease is the outcome of a few critical junctures, referred to here chronologically as “early” and “late” junctures.

Three early critical junctures in particular forced biomedicine to become reliant on animal experimentation—two were established by U.S. laws (1938, 1962) and the third resulted from a catastrophic investment decision (2006) by NIH, the federal agency in charge of scientific research.

Fortunately, two critical junctures emerged of late to set the stage for advancing a humane agenda in science, cutting waste in research and liberating the drug discovery process from its restrictive, animal-centric shackles. Importantly, these recent junctures made it possible to finally break the path dependency associated with animal testing, likely for good. In practical terms, these events rescued the biomedical research enterprise from the disadvantages it endured for decades because of special interest groups and the complacency of many regulators.

The first of these late junctures is defined by the passage of the FDA Modernization Act 2.0 (FDAMA 2.0), a U.S. law since 2022 (Figure 1, no. 5). The second late juncture is a bundle of coordinated policy decisions in 2025 aiming at phasing out animal testing. This pivot towards animal-free science took place at key U.S. health and regulatory agencies, namely FDA, NIH, EPA and CDC (Figure 1, no. 6) as well as the U.S. Departments of the Navy (DON) and Veterans Affairs (VA). A summary of the changes is provided in Table 1, with a full analysis presented chronologically in section 7—“Breaking path dependency in 2025”.

**Table 1:** Coordinated events at U.S. health agencies in 2025 underlying the pivot away from animal testing in drug development and preclinical research.

A pivot away from animal testing proclaimed at U.S agencies in 2025
**April 10—FDA Announces Plan to Phase Out Animal Testing Requirement for Monoclonal Antibodies (mAbs) and Other Drugs.**
FDA Roadmap established a three- to five-year goal to make animal studies the exception rather than the norm. It also provides incentives to drug sponsors using NAMs, including fast-track reviews. FDA would on December 2nd issue a draft guidance for Industry to streamline nonclinical mAbs safety studies.
**April 10—EPA reinstates plans to phase out animal testing.**
EPA reinstated plans to phase out animal testing in chemical and pesticide safety assessments. This move brings back a policy established during the first Trump administration in 2019 and aims to reduce mammal testing by 30% by 2025 and eliminate it completely by 2035, transitioning to NAMs. The plan was suspended under the Biden administration.
**April 29—NIH announces plans to bolster human-relevant tools, reduce animal testing** *.*
NIH's new initiative aims at expanding innovative, human-based science while reducing animal use in research. NIH's plan includes the establishment of the Office of Research Innovation, Validation, and Application (ORIVA).
**May 4—NIH announces eliminating all beagles' experiments on NIH campus*.***
NIH Director Bhattacharya told Fox & Friends in an interview on May 4, “We got rid of all the beagle experiments on the NIH campus”. *
**May 29—Secretary of Navy terminates all DON testing on cats and dogs.**
Navy Secretary announced the termination of all research testing involving cats and dogs within the Department of the Navy. And directed the Surgeon General of the Navy to conduct a thorough review of all medical research programs to guarantee that the research aligns with established ethical guidelines, demonstrates genuine scientific necessity, and adheres to the Navy's fundamental values of integrity and readiness.
**June 23—VA Ends Medical Research on Primates*.***
The Department of Veterans Affairs ends its research involving monkeys. VA has no studies using cats. It has an ongoing study on melanoma that involves dogs.
**July 4—NIH institutionalizes exclusion criteria not allowing animals models in certain Funding initiatives.**
NIH began institutionalizing exclusion criteria disallowing animal models in certain funding opportunities [e.g., Autism Data Science Initiative (ADSI)].
**July 7—NIH eliminates support dedicated to developing solely animal models.**
NIH announced that all new Notices of Funding Opportunity (NOFOs) that relate to animal model systems must now also support human-focused approaches such as clinical trials, real world data, or new approach methods (NAMs).
**Sept 25—NIH establishes a dedicated organoid development center.**
The Standardized Organoid Modeling (SOM) Center will develop standardized organoid-based NAMs that deliver robust, reproducible, and patient-centered research findings.
**Sept 30—NIH announces the “Complement-ARIE Reduction to Practice Prize”.**
This prize challenges research teams to develop and implement human-based biomedical research to reduce the use of animal experimentation. Winners will share a total cash prize of $ 7 million.
**Nov 21—CDC ends all research on primates.**
First reported in *Science* magazine, the CDC will phase out all their research on primates—around 200 macaques are used at the centers mostly for HIV studies. Notably, four decades of HIV research did not produce one single marketable HIV vaccine.

1. In addition to declared initiatives at U.S. Agencies to bolster NAMs, global unions and organizations (e.g., EU, WHO, EFPIA) have ongoing plans to either phase out animal testing or significantly curtail animal experimentation. For example, The World Health Organization (WHO) is actively involved in promoting the development and adoption of NAMs for food safety risk assessment, given their potential to enhance global food security.

^*^Some stakeholders expressed concerns regarding the veracity of several NIH announcements. For instance, White Coat Waste (WCW) posited that testing on Beagles had ended on NIH grounds long before the 29th of April announcement, and that NIH continues to fund new projects utilizing dogs, contrary to what many presumed. WCW has filed lawsuits against the NIH over these matters.

## 3 The beginning: triggering path dependency in 1938

The first critical juncture that triggered path dependency in drug development can be traced back to 1938 with the enactment of the abovementioned Federal FD&C Act. In the prior year, a poisoning incident (known as the Elixir Sulfanilamide incident of 1937) was sufficient to push lawmakers into accepting animals as the “guinea pigs” in testing toxicity of experimental drugs (2–4). Such requirement was incorporated in haste in the newly minted Act, which also gave near unlimited authority to FDA to oversee the regulation of foods, drugs and cosmetics in the United States.

In retrospect, the caveat was that animals are poor predictors of the human response and were never validated as surrogate for humans in toxicity studies. At the time, our understanding of the molecular underpinnings of disease evolution was also limited (5). Today, an objective examination of the many artificial animal models used in safety and efficacy assessments, including NHPs, reveals radical structural, physiological, pharmacological, immunological, digestive, genomic, anatomic, metabolic, and behavioral differences among species (6–8).

To be clear, animals have markedly different, even misleading, toxicity patterns compared to humans (9–16). They provide little de-risking value were they to be the safety gatekeepers of the human response. The toxic chemical behind the 1937 Sulfanilamide poisoning cases (Diethylene Glycol, similar in its composition to modern-day antifreeze) can be found harmful using almost every credible animal-free toxicology methods, including relatively old ones like colorimetric assays. And while Elixir Sulfanilamide tragically poisoned around 100 people in 1937, Vioxx, an arthritis drug approved by the FDA in 1999 and cleared in preclinical stages using animals caused a disaster—the death thousands of individuals by 2005 (17, 18).

The roster of deadly FDA-approved drugs includes the antidiabetic medication Troglitazone (Rezulin) (19), the anti-inflammatory drug Valdecoxib (Bextra) (18, 20) and the pain medication Bromfenac (Duract) (21). In actuality, a Yale university study found that one-third of new drugs on the market had major safety issues years after the medications were made widely available to patients following FDA approvals (22).

Here, we emphasize that even tests directly in humans may not always detect toxicity or predict efficacy. And it is reasonable to assume that some INDs advancing based on preclinical assessments using NAMs will fail in trials as well. Indeed, NAMs must not be viewed as an error-proof system in drug development (discussed also in sections 9 and 10) but a derisking approach that will improve the overall process. Just doubling the success rate, and cutting the costs and development time by even 30% would be, financially and in terms of lives saved, a giant leap forward.

## 4 Fortifying path dependency in 1962

### 4.1 The Kefauver-Harris amendments

The second “early” critical juncture occurred with the passing of the Kefauver-Harris Drug Amendments to the Federal FD&C Act in 1962. An experimental drug must now show efficacy and be examined for side effects—also in animals—before it can proceed to clinical trials. The trigger for such requirement was, in turn, another international public health outcry after the drug Thalidomide, prescribed to pregnant women for morning sickness, caused birth defects in the 50s and 60s (14, 23). But again, Thalidomide does not cause birth defects when given to pregnant rats and mice, the most used laboratory animals (14). So, scores of similar classes of drugs would be erroneously deemed safe and their harmful effects missed using animal screening.

The Kefauver-Harris Drug Amendments related to efficacy standards alongside the 1938 rule seeking to establish safety standards through animal testing were sincere yet misguided attempts at establishing rigor. Ostensibly, they are prime examples of crisis-driven regulatory panic. And regrettably, their main accomplishments as knee-jerk legislation were to shape drug development into the inefficient paradigm it is today.

To the regulatory health agencies, adopting animals to be the “gold standard” was an attractive proposition, especially in 1962. It enabled administrators to “kill two birds with one stone”: (i) make the alarmed public believe that drug safety is now under control, and (ii) bolster the interest of the pharmaceutical industry of which the discovery portfolio at the time was increasingly focused on developing robust animal research pipelines. As a reminder, the prestigious U.S. National Academy of Sciences (NAS), reflecting the sentiment of the academic elite, was promoting as early as 1960 “Animal Models in Drug Development” as the new frontiers of biomedicine. Academic and research institutions, publishers, nonprofit organizations, medical foundations and biomedical trade organizations all contributed to the frenzy surrounding animal testing.

Once animal tests became required by law for efficacy assessments of new drugs, animal testing became standardized and internationally harmonized. Indeed, largescale regulatory standardization began in the sixties with regulatory agencies as well as bodies like the OECD incorporating animal tests into formal guidelines. While beneficial to a smooth operation, change inefficiency multiplied further with international harmonization when stakeholders [e.g., OECD, FDA, EMA (the European Medicines Agency)] aligned animal protocols. And once globalized, reform required cumbersome international coordination, slowing disruption even more. The outcome of all that is animal models becoming the regulatory “gold standard”. Deviation from such doctrine became legally risky, even if better science existed. Universities taught animal models as required methods, textbooks codified them and accreditation bodies required their inclusion. Future scientists were trained into a pre-locked system extending to career lock-in, where tenure systems rewarded animal-based publications, grant panels were dominated by animal-model researchers, and academic prestige typically aligned with animal work output.

### 4.2 The establishment of the national primate research centers (NPRCs)

1962 saw also the establishment of several primate research centers. These serve as breeding facilities of monkey colonies maintained for testing and experimentation on NHPs. Animal researchers convinced the U.S. government to foot the bill for those costly infrastructures, a trend that continues to this day. The National primate research centers (NPRCs) and related “resources” evolved into an expansive network of no less than fourteen facilities across the United States and its territories.

In 2015, NIH stopped supporting research using captive Chimpanzees after they were listed as endangered species by the U.S. Fish and Wildlife Service, but funding continued unabated for research using other NHPs. In fact, a two-part report released by the NIH Office of Research Infrastructure Programs (ORIP) in September and December 2018—i.e., prior to the COVID-19 pandemic and the ensuing NHPs shortage—advocated for an increase in U.S.-based colonies for NHPs. On May 4, 2023, a new report (24) by NAS concluded that supply of monkeys for research is “at a crisis point”. The report, entitled, “State of the Science and Future Needs for Nonhuman Primate Model Systems”, was commissioned by the NIH in response to a congressional inquiry and calls from animal research enthusiast groups.

The 2018 NIH-ORIP report advocated for expansion of U.S.-based colonies of NHPs. The 2023 NAS report echoed, almost exactly, the positions of the NHPs user industry and stakeholders on the issue. Specifically, regarding the quest for expanding U.S.-based breeding programs, the recommendations are very similar to those in the 2018 OIRP report. The juxtaposition of the two reports suggests a dogmatic and stubborn view by the establishment on the issue of NHPs in medical testing. In 2023, we analyzed the NAS report and identified its flaws and inconsistencies (24).

Of note, the trade of NHPs is fraught with the danger of zoonotic transmission (from animals to humans) and the spread of infectious diseases. Demand for NHPs has also driven up criminal activities, including the smuggling of primates to the U.S. Several criminal indictments were handed down by the justice department in recent years related to NHPs smuggling rings into the U.S., motivated by greed.

It is also worth noting that, despite being considered closer to humans than other species, NHPs can be misleading in predicting human disease response, for instance, to COVID-19 (25, 26). Case in point, NHPs did not readily replicate the rapid clinical deterioration or severe acute respiratory distress syndrome (ARDS) seen in humans (26). Generally, no fatalities from disease burden were also registered in SARS-CoV-2 infected NHPs, making them inappropriate for modeling the systemic pathological failure, a hallmark of COVID-19-related deaths (27, 28).

Recently, FDA took a progressive step to phase out animal testing for monoclonal antibodies safety assessments, which are mainly done using primates. This brought to the forefront the predicament of using these sophisticated animals in research. FDA cited the poor reproducibility of data from laboratory primates and the existence of Alternatives (29). Many groups, including the Center for a Humane Economy (CHE, Supplementary Material 1), Animal Wellness Action (AWA), People for the Ethical Treatment of Animals (*PeTA*), Physicians Committee for Responsible Medicine (PCRM), Rise for Animals, Humane World for Animals and White Coast Waste (WCW) as well as elected officials and members of Congress (personal communication) are now calling for sunsetting federal funding for NIH-funded NPRCs.

In short, the National Primate Research Centers (NPRCs) played a key part in fortifying path dependency. The lucrative primate business and the marketing savvy of the stakeholders involved in this trade must not cloud anyone's judgment regarding their dispensability in research.

## 5 Locking-in path dependency in 2006

The third critical juncture, and arguably the most damming is a multibillion-dollar initiative by the National Institutes of Health (NIH) to create thousands of mouse models and make them available to study human diseases (30, 31). The project, initially labeled the Knockout Mouse Project (or KOMP), evolved later to incorporate other styles of genetic alterations. It envisioned a deletion in every gene in the mouse genome—or roughly 18,000 genes.

To begin with, genomic DNA sequences are 85% similar between mice and humans but functional gene expression (the indicator of biological function) is only 50%–60%. Moreover, many genes have varying functions and/or activation patterns at different developmental stages (e.g., embryonic vs. adult) (15). Eliminating a gene at the embryonic stage can produce, in those that survive, markedly altered physiology. Furthermore, gene alterations in mice have long been known to produce dissimilar sequelae and characteristics (called phenotypes) to those presenting in humans—the loss of the cardinal tumor suppressor gene p53 is a prototypical example where tumors unrelated to human cancers arose in mice with p53 loss (32, 33).

Besides, gene regulation, recombination, silencing, redundancy, compensation and strain variance among the same mouse species, let alone inter-species variability, are all critical aspects to consider before producing a single genetically altered mouse model, let alone thousands. In fact, the “kitchen-sink” approach taken by the NIH at the time can be viewed as an example of administrative hubris—health agencies appropriating exorbitant amounts of taxpayer money and spending it on legacy projects. Concomitantly, NIH program level funding swelled from $17.84 to $49.183 billion between FY2000 and FY2023. Still, this is not the most concerning or wasteful damage of all.

Despite strong preclinical efficacy in genetically modified systems, translational failure has been extensive. Case in point, anti-TNF strategies failed to improve mortality in human sepsis trials; most anti-amyloid agents failed to produce durable cognitive benefit in Alzheimer's disease; and many neuroprotective and oncology candidates exhibited substantial attrition during clinical development. Even in cases of apparent translational success, such as PARP inhibitors or targeted oncologic agents, genetically engineered models often have distorted pathways, thereby contributing to high late-stage attrition and constrained clinical applicability.

The irreparable harm caused by this large-scale program is reflected in the massive dissemination of thousands of artificial mouse models with little relevance to human biology. While it is impractical for most laboratories to perform research on dogs or primates, this initiative made it potentially feasible for any laboratory to maintain a rodent colony. This act flooded the market with aberrant study models and saturated the literature with misleading data (5, 7, 34). Indeed, NIH-funded rodent models churned up thousands of publishable yet irreproducible preclinical studies, which are, in principle, the bedrock of drug target discovery. Many such models ended up being qualified as Drug Development Tools (DDTs) by the FDA to “aid” in the safety and efficacy assessments of new investigational drugs and vaccines, further exacerbating the challenge (discussed later).

The focus on animal research at NIH for decades sent investigators on wild-goose chases and normalized—even glamorized—the use of animals in scientific experiments as the supreme, “*in vivo*” science. This animal-centric research culture has contributed to the “reproducibility crisis” and the perceived diminished credibility overall in published studies. Francis S. Collins, the longest-serving former director of the NIH (2009–2021), wrote in the journal Nature in 2014 that “preclinical research, especially work that uses animal models, seems to be the area that is currently most susceptible to reproducibility issues”. (34) Consistently, 89% of preclinical studies, most of which involve animals, cannot be reproduced!

Oddly, it was under Dr. Collins's leadership that the NIH saw the largest expansion of its animal-heavy programs, including KOMP, for which Director Collins was a founding member. As mentioned earlier, KOMP has evolved after many program expansions (e.g., knockout, transgenic, CRISPR), into a massive conglomerate referred to now as KOMP2 and is part of an International Mouse Phenotyping Consortium (IMPC) (35). KOMP initially launched in 2006 during Dr. Zerhouni's tenure as NIH director.

When concerned scientists warned against the dependency on animal testing and betting on the future of discovery on research in rodents, these voices were belittled, discredited and swiftly dismissed by the establishment as anarchists, animal welfare nuts or fringe scientists.

In brief, the NIH-funded endeavor that launched in 2006—thriving and well today—locked in path dependency (Figure 1, no. 4) and fueled the “reproducibility crisis”, which is often referred to poignantly as the “credibility crisis” in scientific research (36–40).

NIH drove the massive build-out of specific animal models, breeding colonies, and genetically engineered mice (including consortia). The agency also facilitated their deep integration into NIH grant structures, training, and study sections. But one must note that NIH also developed as early as 1960s human-based systems and infrastructure aimed at promoting human-relevant research. Notable examples include: (1960s) Clinical research infrastructure (GCRC, Clinical Center), (1970s) Population-level data systems [SEER, (Surveillance, Epidemiology, and End Results) and NIGMS (National Institute of General Medical Sciences) Human Genetic Cell Repository (Coriell Institute)], (1980s) Human biospecimen systems [CHTN (Cooperative Human Tissue Network)] and the National Center for Biotechnology Information (NCBI)—NIH/NLM, (1990s) Information & genomics infrastructure (PubMed, GenBank), (2000s) clinical trial transparency & digital platforms (ClinicalTrials.gov) and the Human Genome Project.

## 6 Disrupting path dependency in 2022

### 6.1 The FDA modernization Act 2.0 became U.S. law

In 2022, a group of legislators concerned by the inefficient drug discovery paradigm but also the out-of-control proliferation of animal use in research shepherded the FDA Modernization Act 2.0 (FDAMA 2.0) in the U.S. House and Senate (41). FDAMA 2.0 became U.S. law later that year. In essence, with this critical juncture (Figure 1, no. 5), lawmakers overturned a century-old rule requiring animal testing to be performed for every investigational new drug or vaccine. Alternatives can now be used *in lieu* of animal tests if they provide sufficient insights in clarifying the safety and efficacy of tested compounds.

FDAMA 2.0 was a landmark legislation and a turning point. It accomplished three important outcomes: first, it made structural modification to the letter of the law. Specifically, the term “preclinical tests (including tests on animals)” was replaced with the term “nonclinical tests”. It also replaced the term “animal models” with nonclinical models. A clear definition of nonclinical tests was also provided in the legislation, to encompass *in vitro* and *in silico* methods, including technology-driven alternatives collectively referred to as NAMs, broadening the definition of nonclinical tests. Second, FDAMA 2.0 underscored the need for human relevancy in conducted research. The new law intended to encourage the adoption of human-relevant testing methods to better predict responses to drugs in people. And third, FDAMA 2.0 encouraged a humane approach to scientific investigations. The new law was also intended to bolster the principles of the 3Rs (Replace, Reduce, Refine) with a special emphasis on the replacement of animals with sufficiently validated, scientifically relevant, and human-specific alternatives. The widely adopted principles of the 3Rs are foundational principles for ethical and scientific responsibility in animal research, first proposed by William Russell and Rex Burch (1959) in “*The Principles of Humane Experimental Technique*”.

### 6.2 The FDA modernization Act 3.0 became a necessity after stonewalling by FDA

The FDA was forthcoming about the fact that 92% of drugs advanced based on animal testing fail to meet the standards for human use, and this rate is growing, not improving (42). Paradoxically, FDA was evasive in updating its regulations to implement the broad policy mandates of FDAMA 2.0 or providing regulatory clarity on the new law. Such failure to act on the part of the agency chiefly responsible for implementing drug laws was a good example of government discordancy, if not malfeasance. To be clear, delays at the FDA vis-a-vis updating the regulations to conform with FDAMA 2.0 came from the previous FDA administration under Commissioner Robert Califf, not Marty Makary.

In 2023, a bipartisan group of Senators, led by Rand Paul, R-Ky., and Cory Booker, D-N.J., sent a demand letter (Supplementary Material 2) to the FDA seeking an explanation for the stultification and implementation timeline of the enacted law. When no progress materialized because of FDA stonewalling, lawmakers guided the U.S. House and Senate to take further decisive actions to ensure the implementation of FDAMA 2.0 (43).

**Figure 2:**
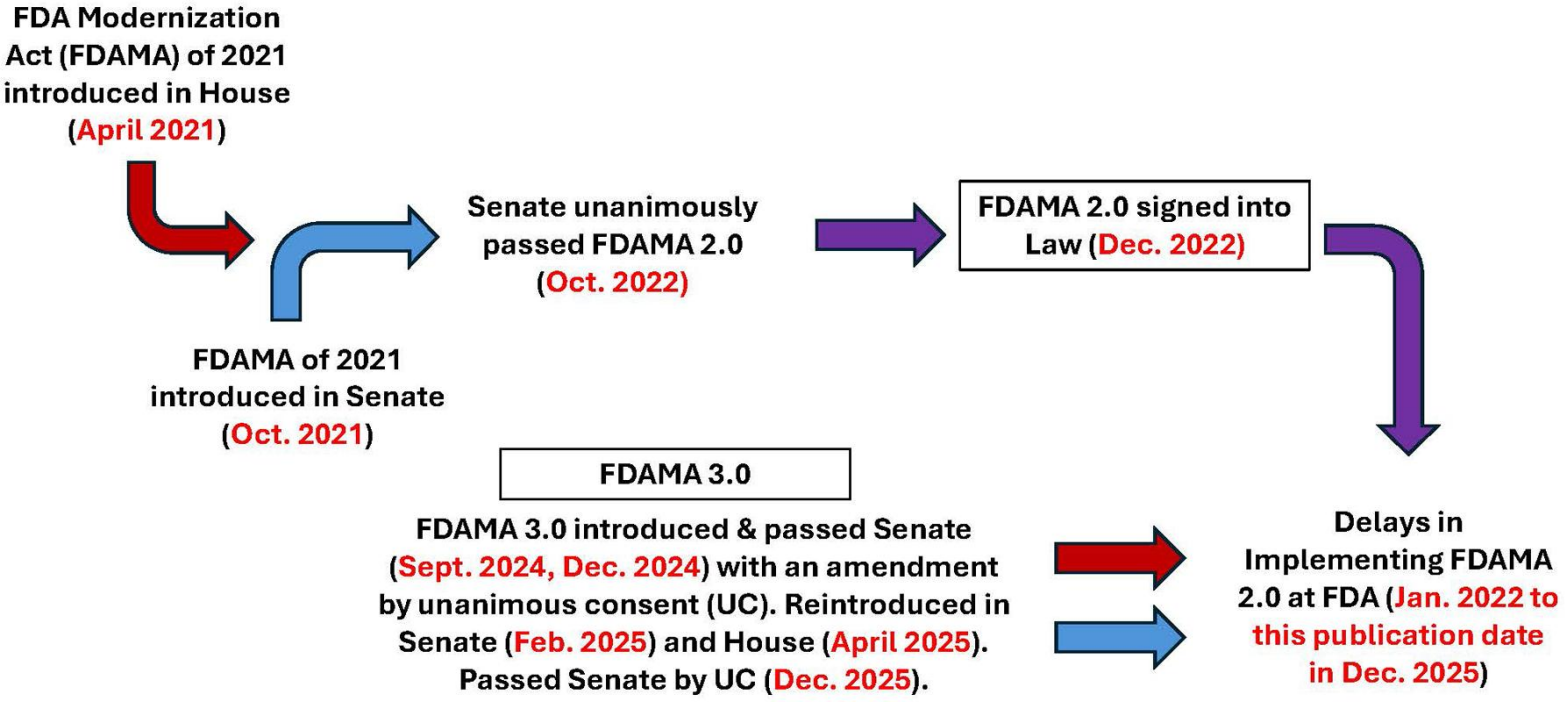
A map chronicling the legislative progress related to FDAMA 2.0 and 3.0 in the U.S. House and Senate.

***Action in the House***: A bill was introduced in the U.S. House on February 6, 2024 (FDA Modernization Act 3.0, H.R.7248) requiring the FDA to establish a process that supports nonclinical testing methods for drug development that do not involve the use of animals (Figure 2). That bill also stipulates that the FDA must establish a pathway by which entities may apply to have nonclinical testing methods approved for use in a particular context. Such qualifying methods must be intended to replace or reduce animal testing and to either improve the safety and efficacy of nonclinical testing or reduce the time to develop a drug. In addition, FDA must issue its decision within 180 days of receiving an application. The FDA must also prioritize the review of applications for drugs that are developed using an approved nonclinical testing method. The FDA must also annually post a report on its website that summarizes the results of the bill's implementation, including the number of applications received, types of methods that were approved, and the estimated number of animals saved as result of these methods.

With the new Congress (119th, 2025–2026), a revised bill was introduced on April 10, 2025, a date that will also be remembered for the historic announcement by the newly appointed FDA commissioner to phase out animal testing requirements for certain classes of drugs. While the initial House version (i.e., H.R 7248), incorporated provision for qualifications and reporting requirements, these elements were dropped from the revised House bill (H.R.2821), which still requires the Secretary of HHS, acting through the Commissioner of FDA, to publish a final rule relating to nonclinical testing methods and the implementation of FDAMA 2.0.

***Action in the Senate***: On December 12, 2024, the Senate unanimously passed the FDA Modernization Act 3.0 (S.5046), a modified version of the House bill (also without the qualification piece and the reporting requirement), demanding that the FDA execute the full implementation of FDAMA 2.0. This bipartisan legislation was re-introduced with slight modifications in the 119th Congress as FDA Modernization Act 3.0 (S.355) in February 2025. On December 16, the U.S. Senate passed S. 355 with unanimous consent (Figure 2), affirming its unshakable desire for a transition away from animal testing and ensuring full Implementation of the FDAMA 2.0 mandates.

The hostility of the previous FDA administration to the FDAMA 2.0 and 3.0 is hard to explain. It is possible that FDA leadership at the time perceived that no reforms to the FDA are needed. It can be argued that a fundamental flaw at the agency is its refractory, dismissive attitude towards outside input or reforms, even those originating from its employer, the U.S. Congress. Indeed, an example of that is the FDA's perfunctory conduct towards the FDA Modernization Act 2.0, a U.S Law since 2022.

In stark contrast, the current FDA administration's attitude towards these reforms is marked with professionalism. The FDA roadmap announced on April 10, 2025 made several references to the FDAMA 2.0 and 3.0, fully embracing the spirit of these reforms.

Organizations like the Center for a Humane Economy (CHE) and Animal Wellness Action (AWA) that championed FDAMA 2.0 and 3.0 and assembled a broad coalition and bipartisan support continue to be unwavering in their advocacy efforts until the full, clear, and indivisible implementation of the law is accomplished. It is expected that the FDAMA 3.0 (all be it in its revised version) makes its way to become U.S. law in 2026, and that regulations related to FDAMA 2.0 will be updated in a manner that conforms with the statute.

Remarkably, in an interview that first aired on December 7 on *Full Measure with Sharyl Attkisson, a political affairs and Sunday news program,* FDA commissioner Makary asserted the agency's commitment to FDAMA 2.0, stating that “*We are going to not only conform, we are going to exceed all of the expectations in reducing animal testing. We are seeing drug applications come in now where they have an outline for animal testing, and we are telling them, we do not want you to do this animal testing. We do not think it is going to inform us about the drug safety - use computational modeling and other technology instead.*”

## 7 Breaking path dependency in 2025

### 7.1 A historic pivot at U.S. regulatory agencies (FDA, EPA and CDC)

On April 10, 2025, FDA commissioner Marty Makary, and only ten days since assuming office, released a “Roadmap to Reducing Animal Testing in Preclinical Safety Studies”. This giant step towards improving human health and modernizing the agency was accompanied by a specific action—replacing safety assessment for monoclonal antibodies (mAbs) done in animals with more effective, human-relevant methods. The choice of mAbs as a starting point is expanded on in Table 2.

**Table 2:** Ten reasons why FDA prioritized phasing out safety assessments in mAbs development.

On FDA Decision to Phase Out Animal Testing for mAbs Assessments
**Therapeutic relevance and immune system engagement**. mAb-based therapies belong to the category of immunotherapy, which can be very powerful in leveraging our own immune system to combat diseases. This includes the area of antibody-drug conjugates (ADCs) (62). Of note, immune responses are triggered in almost every serious illness (e.g., cancer, infectious diseases). Proper functioning of the immune system is also part of our everyday response to stressors. **Support for vaccine enhancement and optimization**. mAbs, while not vaccines themselves, can intensify and diversify the response to vaccines, prolonging their conferred protection and durability. As mAb-based therapies are bolstered in time and quality by the new FDA policy, the outcomes can be especially helpful for individuals with weakened immune system needing more therapeutic options. **Demonstrated failure of animal models in development**. Decades of experience of antibody development using large animals have been disappointing and generally misleading. Sponsors are also realizing that they are prematurely discarding scores of potentially lifesaving drugs just because they fail in animals. **Versatility as a standalone or combination therapy**. mAbs are underscored by their flexibility of use. They can be deployed alongside a wide array of invasive and non-invasive therapies. Their versatility makes them a priority area and a clever choice for this program. For instance, antibodies can be used before (neoadjuvant) or after (adjuvant) a primary cancer treatment to help prevent recurrence, reduce side effects or improve outcomes. **High potential for widespread public health impact**. Dozens of companies worldwide are actively developing hundreds of mAb-based therapies for a wide range of diseases (e.g., Cancers, Cardiovascular, Neurodegenerative, infectious diseases). Rapid advances in this area will likely create significant breakthroughs, fast. It will also encourage collaborations and promote healthy competitions, especially from small biotech companies avoiding animal testing because of cost, high-failure rate or ethical considerations. **Established scientific knowledge and development history**. mAbs have been around for more than two decades. In other words, these treatment modalities are neither based on outdated technologies nor founded on very novice ones (e.g., Crisper-based therapy that are still in early development). As such, there is considerable institutional memory' and know-how in developing antibodies. Many lessons learned over the years by sponsors and regulators make mAbs a good “beachhead” for this FDA program. **Compatibility with artificial intelligence and modeling**. Given the structural, 3D nature of antibody design, mAb-manufacturing benefits greatly from artificial intelligence applications in refining the topography and improving the dimensional precision antibodies for better interaction with a target's complex and dynamic structure. **Specificity of targeting and treatment outcome improvement**. Ab-therapies are targeted and tailored as they seek to modify one defined and specific target (e.g., a cell surface receptor, a circulating protein) causing a disease or an abnormality. Any advances there triggered by this new FDA rule will lead to therapies with less side effects and more precision. **Cost-efficiency compared to animal-based testing**. Large complex mammals, aside from unreliability to model human diseases, are cost prohibitive. Antibody testing typically requires 150 primates (av. $50,000 each), so a cohort will cost $7.5 million. Studies showed that this can be achieved with a fraction of the cost using non-animal testing methods. For instance, a cost comparison analysis by the Moderna showed that an experiment using animal-free OOC cost $325,000 compared to $5.25 million using NHPs, with experimental time reduced several folds when using OOCs. **Avoidance of misleading inter-species variability**. Today, on average 92% of experimental drugs fail clinical trials after animal data justified their advancement to the clinical stage. The amount of waste in this process is incalculable. In monoclonal antibody testing, reducing the reliance on animals (poor predictors of safety and efficacy responses in humans) and shifting to human-relevant models instead will improve experimental outcomes.

The roadmap established an ambitious three- to five-year goal to make animal studies the exception rather than the norm for preclinical testing. Importantly, it provides incentives to drug sponsors, such as fast-track reviews, for using animal-free methodologies. The roadmap also took the position that once 21st-century technologies grounded in human biology replace animal testing, consumers will see a drop in drug prices, more treatments and cures for patients in crisis and fewer side effects from drugs.

“For too long, drug manufacturers have performed additional animal testing of drugs that have data in broad human use internationally. This initiative marks a paradigm shift in drug evaluation and holds promise to accelerate cures and meaningful treatments for Americans while reducing animal use”, said Dr. Makary. “By leveraging AI-based computational modeling, human organ model-based lab testing, and real-world human data, we can get safer treatments to patients faster and more reliably, while also reducing R&D costs and drug prices. It is a win-win for public health and ethics” (45).

In an interview on April 18, Makary elaborated on his consequential decision asserting that “he wants to see as much reduction in animal testing as humanly possible” (46). According to another FDA statement explaining the new move, “the new approach is designed to improve drug safety and accelerate the evaluation process, while reducing animal experimentation, lowering research and development (R&D) costs, and ultimately, drug prices”. Of note, on December 2, FDA released a draft guidance on reducing testing in NHPs for Monoclonal Antibodies as a valuable follow up to the April announcement (44).

Furthermore, in what appears to be a coordinated effort, EPA Administrator Lee Zeldin affirmed On April 10 as well his plans to phase out animal testing for chemical safety screening. Andrew Wheeler, the agency's head at the end of the first Trump term, had set deadlines to reduce mammal testing by 30% by 2025 and eliminate it completely by 2035. The plan was suspended during the Biden administration but is now being revived, with a broadening of scope to include all vertebrate animals, not just mammals.

The most recent policy shift came from the Centers for Disease Control and Prevention (CDC). On November 21- and reported with a title “*Exclusive: CDC to end all monkey research*” in *Science* magazine—CDC is set to terminate all their research on primates—around 200 macaques are used at the centers mostly for HIV studies. Decades of HIV research on primates did not produce any marketable HIV vaccine—one of the most challenging areas using animal models.

### 7.2 Unprecedented steps towards animal-free science at NIH

Two weeks after FDA commissioner and EPA administrator announced the FDA roadmap and the intent to phase out animal testing at EPA, the new NIH director, Jay Bhattacharya, in turn, rolled out his plan to prioritize human-based research and reduce the use of animals in NIH-funded research (47). Importantly, this rollout on April 29 was supported also by specific actions including the establishment of a dedicated office at the NIH named the Office of Research Innovation, Validation, and Application (ORIVA), and the termination of all beagle experiments on NIH campus.

“For decades, our biomedical research system has relied heavily on animal models. With this initiative, NIH is ushering in a new era of innovation”, said NIH Director Dr. Jay Bhattacharya. “By integrating advances in data science and technology with our growing understanding of human biology, we can fundamentally reimagine the way research is conducted—from clinical development to real-world application. This human-based approach will accelerate innovation, improve healthcare outcomes, and deliver life-changing treatments. It marks a critical leap forward for science, public trust, and patient care” (47).

Subsequently, NIH committed additional steps for reducing the reliance of NIH funded research on animals. This encompasses excluding the use of non-human models from certain funding initiatives (e.g., the Autism Data Science Initiative) as well as eliminating funding for proposals solely developing animal models (Table 1). Overall, plans to refocus the agency on cutting-edge advanced technologies (e.g., AI, advanced cell models) are poised to re-establish the NIH as the world's trusted leader of innovation-driven research and discovery.

Despite tangible progress, NIH has more work to do towards advancing Alternatives and reducing its reliance on animals. For example, the agency has not addressed its unabated funding for the National Primate Research Centers (NPRCs) or its expansive animal phenotyping consortium, two of the critical junctures that shaped path dependency as posited in this analysis. In response to a request from CHE and AWA to downmodulate funding for NPRCs, redirecting funds to the qualification and development of human-relevant NAMs (Supplementary Material 1), the agency's reply in May 2025 was less than encouraging (personal communication). Furthermore, the agency continues to promote its signature program on NAMs as “Complement-ARIE” or Complement-Animal Research in Experimentation. Previously, we cautioned against making NAMs mere “complements” to animal experimentation and the dire effects this would have on torpedoing the principles of the 3Rs (48). This aspect, given its importance, is further discussed under Section 10—“Challenges facing the momentum of animal-free testing”.

NIH director Bhattacharya has been crystal clear in articulating his position on animal research, NAMs and how to “fundamentally reimagine the way research is conducted”. But hyperbolic pronouncements and an admixture of confusing narrative have diminished the enthusiasm in NIH's resolve to implement its original and bold pledges.

On August 15, NIH further outlined its strategic priorities through a series of broad principles, presented as gold standards, and grouped under Advancing NIH's Mission Through a Unified Strategy (49). As expected, the Unified Strategy reflected many priority items in healthcare on the agenda of the Trump administration with emphasis on areas like nutrition, artificial intelligence, understanding autism, oversight of NIH funding overseas, and HIV/AIDS research to name a few. The topic of “Alternative testing models” was also listed in the NIH's Unified Strategy but is, regrettably, meager and confusing.

Plan documents reveal that “*NIH is establishing the Office of Research Innovation, Validation, and Application [ORIVA] under the Division of Program Coordination, Planning, and Strategic Initiatives, to develop, validate, and scale the use of human-biology-based new approach methodologies (NAMs) **to complement animal models** and enhance investigations*”. Once again, NIH is inexplicably doubling down on the bygone doctrine of using NAMs to “*complement animal models”.* What is conspicuously missing from NIH's plan as well is an emphasis on human relevancy in conducted research, which should be an utmost priority at the agency now, if not its main guiding principle.

Sustained advocacy and earnest engagement with the NIH could help restore missed opportunities for reforms at the agency. Nevertheless, the NIH plan announced on April 29 to bolster alternatives alongside the release of the FDA roadmap to reduce animal testing in preclinical safety studies on April 10 and similar forward momentum at the EPA and CDC represent watershed moments for scientific discovery. These are steps in the right direction towards modernizing U.S. health and regulatory agencies, reducing failure rate in clinical trials, restoring credibility to preclinical research and eliminating inefficiency and waste from the discovery process (29, 47).

### 7.3 Decisive steps towards animal-free science at the U.S. Navy and the VA

On May 29, the secretary of the Navy, John Phelan, announced that he has ordered the Navy Surgeon General to review all medical research programs to ensure they align with ethical practices and “true scientific necessity”. Importantly, the Secretary announced the termination of all Department of the Navy testing on cats and dogs (Table 1), citing the inhumane nature of the practice and the availability of alternative research methods. “It gives me great pleasure to terminate all Department of the Navy's testing on cats and dogs, ending these inhumane practices and saving taxpayer dollars”, Phelan wrote on social media. On June 23, the Department of Veterans Affairs (VA) also took steps towards phasing out animal testing, ending medical research on primates (Table 1). The policy changes described above at key federal agencies fractured path dependency associated with animal testing and made scientific discoveries wide open to new possibilities built on innovation. Decisive leadership was the missing driver to advance reforms. But today, regulatory agencies are stepping up, not only to provide regulatory clarity on the use and adoption of animal-free alternatives but also to spark further innovation in these areas through incentives, initiatives and meaningful collaborations with stakeholders (Table 1), including legislators.

## 8 The immediate benefit of breaking path dependency

Making regulatory policy less dependent on animal testing confers immediate benefit to many challenging issues, including (i) instances of life-threatening and complex diseases with species relevance limitations—in other words, where no animal models exist due to the lack of a pharmacologically relevant comparison to humans; (ii) rare diseases where 95% of the 7000-plus diseases afflicting around 30 million Americans have not a single FDA approved drug (NCATS rare disease factsheet); (iii) in the growing therapeutic areas of biologics, where therapies (e.g., immunotherapy, cell-therapy) are, by design, human-specific—the use of animals there is misleading and scientifically unjustified; (iv) for repurposed drugs or in cases where prior target modulation experience exists; and (v) for mitigating the ethical conundrum of killing around 200 million laboratory animals under the banner of scientific research each year. Incorporating NAMs prominently in research studies is also a significant benefit of breaking path dependency as discussed in detail in the following section.

In October, *Qureator Inc*., a San Diego–based company in the space of AI-powered, human-relevant organ-on-a-chip platforms announced that its IND submission for an oncology combination therapy (the combining anticancer and drug dual-action mitotic checkpoint inhibitor BAL0891with an immune checkpoint inhibitor) was based solely on human vascularized organoid (organoid-on-chip) efficacy data and achieved “first” IND approval under this paradigm—a milestone under FDA Modernization Act 2.0.

Such forward momentum necessitates that regulatory standards must move towards future innovations and technologies, not backwards, recycling limiting models hoping they mimic a human response. Again, the recent example of the FDA on phasing out monoclonal antibody testing for toxicity assessments in primates and the *Qureator* precedent above are signs of real progress. They must be emulated by other agencies, including the NIH, EPA and CDC. It is plausible that a stepwise regulatory process combining NAMs with animal models will emerge at regulatory agencies prior to fully adopting animal-free assessment paradigms for safety and efficacy. Such scenario will likely entail data from one large laboratory animal instead of two as has been the practice.

## 9 A prominent role for NAMs in the path ahead

The FDAMA 2.0 expressly “allow[s] for alternatives to animal testing for purposes of drug and biological product applications”. Alternatives or NAMs are experimental tools, systems or platforms designed to capture with accuracy, cell-to-cell communication, drug interaction, fluid dynamics, physio-mechanical features alongside other structural, dimensional and functional parameters of a physiological system for research and discovery purposes. Their utility as consolidated investigative models are made possible by progress in fields like biophysics, regenerative medicine, materials science and electrical engineering.

Among the most popular NAMs today are organ-on-chip platforms (OOC), organoids and spheroids that use live cells and functional tissues, and generative AI-driven *in silico* models that yield novel insights from patterns learned from massive datasets, including predicting toxicity of chemical structures and biological materials. Large scale studies have shown that NAMs outperform conventional animal models in predicting DILI (or drug-induced liver injury) (10, 11), a key barrier in drug discovery [(also reviewed here (12)]. A comprehensive summary of the advantages of NAMs is provided in Table 3. NAMs are being steadily incorporated into all aspects of scientific investigations across academia, industry and governmental agencies—from pharmaceutical development to environmental health and safety testing to chemical toxicity to food safety and cosmetics research.

**Table 3:** Various contexts in which NAMs confer marked advantage over conventional research models, including animal models.

Advantages of NAMs in Modeling Human Health and Disease
**1. Improve drug safety and the assessment of Monoclonal Antibodies (mAbs),or antibody-drug conjugates (ADC), compared to existing animal-based assessment packages.** According to the FDA, replacing animal testing in the development of monoclonal antibody therapies with more effective, human-relevant methods like NAMs improves drug safety and accelerates the evaluation process, while reducing animal experimentation, lowering research and development (R&D) costs, and ultimately, drug prices. In principle, this extends to antibody-drug conjugate (ADC) used in combination therapy, where a monoclonal antibody can deliver a potent chemotherapy directly to cancer cells (29, 62) **2. Predict the safety of drugs with 87% sensitivity as compared to no more than 50% using existing animal models.** This is vital for producing safer, cheaper, and more effective drugs all-around. It will reduce the high failure rate in clinical trials (currently at 95%), and lower post-market withdrawal and termination of drugs (38, 56, 57) **3. Prevent the ill-advised premature abandonment of scores of potentially life-saving drugs as is often the case using animal data.** Scores of drugs are being discarded prematurely due to misleading safety and efficacy data in animals. The use of MPS has proven its advantage of not falsely labeling safe drugs as toxic with very high specificity [one study reported100% specificity (56)] (38, 56) **4. Provide the capacity for repeated, serial, or sequential testing, including same site re-interrogation in ways that are impossible using other models, including animal models.** Technological advantages intrinsic to microfluidics and MPS platforms have practical implications for designing accurate physiological simulations and generating high-content experimental and therapeutics data. With more progress in improving throughput, MPS can soon deliver unique high-content/high-throughput outcomes (38, 63) **5. Replicate with marked accuracy the unique features of the human gut flora, microbiomes, and characteristics of immunological nature in people.** A striking difference between humans and animals is the function of the microbiome and other species- and gender-specific digestive and immune responses that can be modeled elegantly using NAMs (38, 64) **6. Facilitate the rapid repurposing of countless existing drugs where established target manipulation experience exists.** This is a key advantage when testing the efficacy of repurposed drugs or exploring new indications. A recent example is the FDA acceptance of efficacy data for rare neuropathies using a repurposed antibody, TNT005 (38, 65, 66) **7. Enable the modeling of high-complexity anatomies, pathologies, and conditions traditionally deemed risky, inoperable, and immedicable.** The entanglement of immunity, metabolism, and tissue homeostasis underlies many complex human diseases and phenomena (IBD, BM injury, Angiogenesis). Here, using NAMs is potentially a gamechanger (38, 67, 68) **8. Spark investments in neglected and rare conditions that receive little to no attention due to financial return on investment considerations.** Hundreds of rare diseases with small patient populations are deemed risky. On average, it takes $2.6 billion to bring a new drug to market. In this regard, MPS can reduce the barriers of entry for many startups given the MPS manageable infrastructure needs and the no reliance on costly and unreliable animal models (38, 65) **9. Save critical time during pandemics and other emergencies, including rapid deployment to eliminate poor candidates in vaccine target screening.** Critical for future pandemic prevention and public health emergencies. As an example, MPS platforms were able to predict the poor response of SARS-CoV-2 to Hydroxychloroquine effectively and rapidly (69, 70) **10. Generate productivity gains by reducing the cost and time required to develop new drugs.** NAMs can lead to productivity gains of $24 billion annually according to some industry estimates. $3 billion could be gained annually if Liver-Chips were used to predict liver toxicity across the discovery pipeline (56) **11. Capture the physio-mechanical properties of tissues and organs which are critical for the precise modeling of proper biological functions in humans.** A key feature of advanced NAMs is mimicking the movement of cells, liquids and materials for unique insights into the relationship between kinetics, hemodynamics, signaling, and flow. All essentials in physiological measurements/treatment response (69, 71) **12. Incorporate AI platforms and other technologies like biosensors providing actionable health data in real-time throughout discovery, including during and after treatment.** This is critical for (i) the modern design of effective drugs, (ii) the refinement of therapeutic responses, and (iii) the tracking of molecular outcomes at the granular level. Ideal too for ‘digital twin’ frontier in medicine (38, 72) **13. Lead to new frontiers in personalized medicine based on the very individual's natural history, gender, genetic predispositions, and/or specific response to therapy.** Disparate elements of this are now being tested. Future application of NAMs in personalized diagnosis and treatment using one's own biological materials will be a disruptive innovation in healthcare delivery & services (73–78) **14. Improve global food security given the value of NAMs in safety risk assessment.** Public health and food safety agencies including the WHO see the potentials of NAMs in Next-Generation Risk Assessment and capacity building for low- and middle-Income Countries. **15. Enable the enhanced use of digital twins for simulation, prediction, and optimization.** Digital twins which are virtual representations of physical objects are being used to model patient responses to treatments, personalize medicine, and accelerate drug discovery. NAMs involving AI, machine learning, and cloud computing, are very valuable in this regard.

To this end, advances in NAMs and their application are poised to: (1) improve the safety and efficacy of vaccines and experimental drugs compared to existing models—e.g., in monoclonal antibodies (mAbs) assessments, as FDA posited on April 10 2025, (2) help reduce the failure rate in clinical trials and lower post-market withdrawal/termination of drugs, (3), facilitate the rapid repurposing of existing drugs, (4) prevent the jettison of potentially useful agents prematurely during preclinical testing, (5) improve pandemics preparedness and emergency response by refining therapeutic development, including vaccine production, (6) spark investments in neglected conditions receiving little attention due to financial return on investment considerations, (7) enable modeling of high-complexity anatomies, pathologies, and conditions traditionally deemed risky, inoperable, and incurable, (8) create new study designs for repeated, serial, or sequential testing, including same site re-interrogation in ways that are impossible using any other model, (9) capture the physio-mechanical properties for the modeling of precise biological functions, (10) reduce the economic burden, health disparities, cost and time R&D requires to develop new drugs or protect the environment, (11) lead to new data, roadmaps and legislations in personalized medicine, health economics, chemical and food toxicity, environmental health and safety testing, nutritional safety, planetary health and the development of natural foods, and (12) improve health assessments based on the individual's natural history, gender, genetic predispositions, and/or specific response to therapy.

## 10 Challenges facing the momentum of animal-free testing

### 10.1 Presenting NAMs as “complements” to animal testing

In business strategy, complements are invoked in the context of maximizing competitiveness for a particular industry. More specifically, the “complement” terminology is used in what is referred to as “5 forces + 1 complement” framework, also known as Porter's Five Forces analysis. This framework examines five competitive forces (not discussed here for brevity), plus the added impact of the one complementor to maximize profitability of businesses.

To begin with, the premise of complements is that both entities (animal testing on the one hand and NAMs on the other in this case) offer great value on their own (a highly debatable proposition regarding animal testing) (48). But jointly they can form a unique, rare hybrid that will be hard to imitate, hence the competitive advantage. Cameras on drones are examples of complements, combining digital photography with aviation capabilities.

Effectively, the “complements” narrative will transform the 3Rs principles into a feckless 2R + 1C version—Reduce, Refine and Complement. Besides, in the event the “complements” systems becoming widely used, as intended, extricating one component from the mix then (i.e., removing the animal component) is next to impossible. It will be akin to trying to convince a car manufacturer to sell cars with no breaks.

Making NAMs mere “Complements” to animal experimentation is the modern-day Trojan Horse of the animal- industrial complex to occupy the NAMs space (48). But one must have faith in the sophistication of the legislators and the new NIH director. It is unlikely that they will be duped by the “complement” ruse. Here, it is imperative for leaders at health and regulatory agencies, as part of purging “Group Think” and ridding themselves of “industry capture”, to be deliberate in the way they present and advertise their signature funding programs for NAMs. The issue is not just a nomenclature preference. Agencies have a fundamental obligation to preserve the principles of the 3Rs, prioritizing *Replacement* strategies. This includes preventing their hollowing out by the knavish tricks of the animal-industrial complex. From that perspective, NIH must cease right away promoting its NAMs program as a “complement” to animal in research experimentation.

### 10.2 Disseminating animal-derived NAMs for predicting drug response in humans

A bizarre narrative, oddly sanctioned by prior NIH and FDA administrations, encouraged the introduction of animal-derived NAMs in drug discovery. Agency representatives were openly advancing such plans at scientific conferences, even recommending biotech scientists develop animal-based tools, like murine OOCs. Several technology developers, already tethering by a thread due to financial constraints, are generally vulnerable to “feedback” from regulators and can be co-opted to go along.

The distractions of mass-producing animal-based NAMs to study humans undermine the momentum of late made towards bolstering human-relevant methodologies. This also clashes, particularly today, with the series of policy changes announced in April 2025 at FDA, NIH, EPA and DON seeking to phase out animal testing (29, 47, 50, 51).

An NIH-commissioned report released in December 2023 promoted the development of animal-based NAMs. This report, “Catalyzing the Development and Use of Novel Alternative Methods”, was also the impetus for launching the abovementioned program at the NIH called Complement-Animal Research in Experimentation. In relevant parts, the report stated that it would “build on recommendations by the NIH Advisory Committee to the Director (ACD) Working Group on Enhancing Rigor, Transparency, and Translatability in Animal Research, which were adopted by the ACD, to explore alternative approaches and to improve selection, design, and relevance of animal models”. Consistently, the first recommendation of the December report was to “Prioritize the development and use of combinatorial NAMs (which also includes animal-based NAMs)”.

Animal-derived NAMs are profitable to the animal testing industry, always looking to reinvent itself. But if laboratory animals as living organisms are poor predictors of safety, efficacy and therapeutic response in humans (8, 38), why would tools derived from animal cells and tissues be any better? NAMs designed for modeling human diseases or developing human drugs should be benchmarked against human data, the true gold standard, not compared to artificial animal models, animal-NAMs or any other make-believe surrogates of the human condition. That said, species-specific NAMs can add considerable value and should be encouraged in the context of veterinary medicine research and improving medications designed for animals.

### 10.3 Additional challenges and shared responsibilities

Several behavioral and logistical challenges must be overcome to prevent path dependency from reassembling:

***First, health agencies need to follow through on their commitment to animal-free science***. Public policy reforms must be rendered immune to partisan politics. The EPA example serves as a stark reminder that political conflict can limit progress on Alternatives—e.g., the tabling of an existing phase out plans under a different administration, as describe earlier (52). Furthermore, agencies must proactively engage in promoting Alternatives. In this context, it was refreshing to witness that the recent establishment of the ORIVA at NIH or the roadmap at FDA were voluntary actions—originating within HHS. A recent collaboration to establish a Validation & Qualification Network (VQN) as part of a new public-private partnership to accelerate the deployment and regulatory implementation of NAMs is a good example of a home-grown initiative as well. In the past, activities favoring Alternatives at U.S. agencies were generally “imposed” following inquiries by Congress and reports from the Government Accountability Office (GAO). For instance, the establishment of ICCVAM (Interagency Coordinating Committee on the Validation of Alternative Methods) in 2000, came to fruition only after GAO initiated a report to remedy deficiencies. GAO recommended that HHS's National Institute of Environmental Health Sciences facilitate the establishment of an interagency workgroup to develop metrics for assessing progress on the development and promotion of Alternatives to animal use.
***Second, a robust, high-capacity qualification program for Alternatives must be established.*** Concomitantly, the traditional NIH-FDA loop for churning-up and qualifying animal models (which is consuming considerable resources) must be disrupted. In the existing paradigm, NIH funding creates thousands of animal models. The FDA then qualifies many of these models as Drug Development Tools (DDTs) hoping they can help predict safety and efficacy of drugs in humans. Such perpetual NIH-FDA feed-forward loop, volleying animal tools back and forth has been untouchable, let alone unbreakable, even though it yields a stupefying failure rate averaging 92% in translating drugs from preclinical testing to market. Oddly, this established scheme has been the core of the U.S. national strategy to advance modeling in drug development for decades. The Innovative Science and Technology Approaches for New Drugs (ISTAND) pilot program has been in existence now for several years. It was designed to improve FDA's efforts in this domain and was established after a scathing 2019 report from GAO, in response to a congressional inquiry. But ISTAND is a meager pilot program that anticipates accepting “2–4 submissions each year”. This is incompatible with the spirit of innovation in a competitive capital market. The applications solicited and processed by the FDA on NAMs qualifications must count in the hundreds. ISTAND must evolve into a high-capacity and permanent NAMs qualification program. A robust NAMs qualification program is the “missing tooth” within the three established DDTs at the FDA: Biomarkers, Clinical Outcomes Assessment and the Animal Models qualification programs. It was gratifying to see that FDA made this program permanent in summer of 2025 towards bolstering its function, a change we have advocated for over many years (53).
***Third, academic culture must evolve to become more engaged in developing, utilizing, and refining Alternatives***. Credible and established hubs for Alternatives at academic institutions such as the Wyss Institute at Harvard and The Johns Hopkins University Center for Alternatives to Animal Testing (CAAT) can likely guide this global transition effort in academia towards human-relevant science. Programs within NIH, like NCATs, aiming at accelerating the development, validation, and broader adoption of NAMs should also acquire a more invigorated role in the years ahead. Overall, such change in culture must include (i) a transition plan for researchers (including financial incentives) seeking to shift to more modern Alternatives, (ii) a focus on training the new generation of scientists on Alternatives, and (iii) an effort to revise curricula that continue to disseminate faulty concepts and dogmas built on animal data. Purging erroneous and misleading studies from scientific literature will, in the long run, provide a great service to the scientific discipline. Regrettably, many in the academic culture view funding for Alternatives as a boon to support or supplement their established animal studies and an opportunity for “double-dipping”. Here, establishing clear exclusion criteria within federal funding opportunities to disallow support for non-human methods when appropriate, as NIH commendably began doing in July 2025, is a step in the right direction. We formally urged the NIH Director in April 2025 to implement similar policies (Supplementary Material 3).
***Fourth, standardization and harmonization of standards are critical drivers for the wider dissemination of NAMs***. This technical process establishes common elements that enable data scientists, investigators and regulators to speak a similar language when examining, interpreting and curating results from various NAMs platforms. Entities like OECD, International Organization for Standardization (ISO), European Union Reference Laboratory for alternatives to animal testing (EURL ECVAM), The International Council for Harmonization of Technical Requirements for Pharmaceuticals for Human Use (ICH), FDA, EPA, EMA are all engaged in the standardization of NAMs through technical committees and workgroups. This is an ongoing process given continuous progress in the NAMs field. Of particular importance to drug development is the production of revised documents on Guidance for Industry such as S6 (Preclinical Safety Evaluation of Biotechnology-Derived Pharmaceuticals) or S9 (Non-clinical evaluation for anticancer pharmaceuticals). It has been suggested by industry insiders that “prior experience with modulating the target, and/or the ability to address putative risks using NAMs and/or clinical mitigation and monitoring, should be the triggers for a NAM-based filing irrespective of the availability of a pharmacologically relevant species” (54). Again, this type of guidance, if captured in ICH guidance like ICH S6 or ICH S9 will advance the future state of NAMs in regulatory acceptance, reduce uncertainty and result in greater adoption of NAM-based regulatory filings.
***Fifth, foresight from industry partners and technology developers is needed***. Pharma, biotech, startups and Contract Research Organizations (CROs), especially those heavily invested in animal experimentation must alter their business model and rapidly embrace change. Incentives and rewards should be provided to companies making tractable and earnest efforts towards Alternatives. Notably, IQMPS, an alliance of large pharmaceutical companies, has done important work in this regard. Many biotech companies (e.g., Emulate, InShpero) have also conducted groundbreaking studies to demonstrate how NAMs outperform conventional safety packages. That said, technology developers overall must resist presenting NAMs as the catch-all solutions for every challenge. They have an obligation to disclose the limitations of their tools and methods, including defining their context-of-use and the specific advantage they provide. As itemized in Table 3, NAMs have enormous capacity to revolutionize biomedicine without the deployment of hyperbole.
***Sixth, a prioritization scheme will safeguard a responsible transition to animal-free science***. Reforms to animal testing and the concomitant bolstering on NAMs must proceed in a way that would confer the most benefit like addressing high priority areas and replacing animal testing when feasible. For instance, it has been long known that DILI is a real barrier in the safety assessments of new drugs, so focusing on such aspect will yield considerable returns. It is not a coincidence that studies involving OOC, including some in partnership with FDA, have prioritized DILI studies (55–57). It has also been known through practice that many assays using non-animal methods (e.g., skin sensitization tests) outperform standard *in vivo* animal testing packages and that safety assessments of monoclonal antibodies using primates are not reliable, causing the FDA to switch to NAMs instead (29). Of note, OECD test guidelines were updated in June to allow *in vitro* and in chemico methods (i.e., NAMs) to be used for hazard identification as alternate sources to what was considered, until now, the first-choice method for *in vivo* testing in skin sensitization—murine Local Lymph Node Assay (LLNA) Several frameworks are actively being developed across many testing areas [e.g., Developmental neurotoxicity (DNT)] for the sustainable regulatory implementation of NAMs (58).

Switches on a rolling basis like these will declutter and deconflict the testing space, enabling technology developers and regulators to focus on new pressing areas. Today, these include: (i) instances of life-threatening and complex diseases with species relevance limitations—in other words, where no animal models exist due to the lack of a pharmacologically relevant comparison to humans, (ii) rare diseases where challenges are daunting, including drug approvals, (iii) in the growing therapeutic areas of biologics, where therapies (e.g., immunotherapy, cell-therapy) are by design very human-specific—the use of animals there is misleading and scientifically unjustified and (iv) for repurposed drugs or in cases where prior target modulation experience exists.

## 11 Discussion

### 11.1 Some in the academic elite mislead the legislature on animal testing

It can be argued that members of the scientific elite duped the U.S. Congress twice—once in 1938 and then in 1962 (Figure 1, no. 1 and 2)—into an alliance that rendered the American biomedical research enterprise inorganically linked to experimentation on animals. Forecasting human response using only animals, including large mammals, was presented to lawmakers then as the ideal solution to hedge against health risks, without sufficiently explaining the often-misleading nature of artificial animal models. This includes failing to articulate that most animal models used to study humans have disparate characteristics, with hardly any capturing the sequela of human diseases.

That spurious alliance sought to forcibly fit a square in a round hole. In doing so, it strangled, through statutes nonetheless, any “outside-of-the-animal-box” thinking in discovery research. To that extent, the exclusive reliance on animal testing translates today into irrecuperable delays in the development of medicines, missed opportunities due to misguided regulatory principles and exorbitant costs ultimately passed onto consumers.

Of note, the call to establish serious programs on Alternatives is not a new proposition. In 1979, H.R.4805, a bill introduced in the 96th congress (1979–1980) sought to establish a National Center for Alternative Research. The bill also sought to develop and coordinate alternative methods of research and testing which do not involve the use of live animals, to develop training programs in the use of alternative methods of research and testing which do not involve the use of live animals, to disseminate information on such methods, and for other purposes. In addition, it provided for redirecting between 30 and 50 percent of the total Federal research funds involving the use of live animals to develop alternative methods.

Such a perceptive plan almost fifty years ago was met with extreme resistance, particularly from the NIH at the time. In a refractory response to lawmakers' inquiries, GAO conveyed the dismissive NIH position (Supplementary Material 4). Had leaders of health agencies invested in Alternatives at the time, countless lives would have been saved and the current state of Alternatives would have been markedly more advanced. From that perspective, the policy decisions of April 2025 to bolster Alternatives, while welcomed with enthusiasm, are regrettably half-a-century late.

The disturbing excerpt below summarizing NIH's position in 1980 on Alternatives reads in its entirety like the statements given by NIH officials prior to Dr. Jay Bhattacharya assumed the mantel of the agency in April 2025—that is a stretch of 45 years:

“NIH, in commenting on H.R. 4805, stated that while the intent to promote animal welfare was commendable, legislation of the scope and nature proposed **was both unnecessary and unworkable**. NIH noted that the bill would prohibit the use of funds for animal testing once an alternative is identified. It argued that the results from research and tests involving the use of alternatives are often validated through the use of live animals—an essential step in determining possible effects’ on human health and safety. NIH also said that duplicative research and testing, in which the results of one investigator are confirmed or disproved by another, is an important part of the scientific process. NIH further pointed out that requiring extensive reprograming of funds from live animal research to alternatives would severely limit support for a large number of important research programs and hamper scientific progress in many areas of biomedical research. It added that the amount of funds proposed for developing alternative research methods could not be wisely expended when basic knowledge and technology are lacking.”

### 11.2 Advocacy, technology and decisive leadership made animal testing reforms possible

The failure rate in translating health outcomes from preclinical animal data to the clinic made it clear that the existing paradigm in research and discovery must be disrupted. What remained unclear, until recently, is when and where this change will occur? What would it entail? And importantly, who will take the first step?

“We have moved away from studying human disease in humans. We all drank the Kool-Aid on that one, me included,” said former NIH director (2002 -2008), Elias Zerhouni. “With the ability to knock in or knock out any gene in a mouse—which can't sue us, researchers have over-relied on animal data. The problem is that it hasn't worked, and it's time we stopped dancing around the problem…We need to refocus and adapt new methodologies for use in humans to understand disease biology in humans”.

This elusive “need to refocus and adapt new methodologies” materialized in 2025 in the form of a series of policy decisions by the newly appointed HHS Secretary Robert F Kennedy Jr.'s team, namely the Commissioner of the U.S. Food and Drug Administration (FDA) Marty Makary, the Director of the National Institutes of Health (NIH) Jay Bhattacharya, and the Administrator of the Environmental Protection Agency (EPA), Lee Zeldin. That is addition to decisive actions by the Secretary of the U.S. Navy John Phelan.

FDAMA 2.0 shined the spotlight on the futility of animal testing since 2021 including the availability of feasible Alternatives that outperform conventional animal testing packages across many applications. This critical juncture created by having a U.S. law that removed the requirement for animal testing, provided the trigger needed to advance overdue reforms in discovery science. It can also be argued, ironically, that the resistance to implement FDAMA 2.0 by the previous FDA administration as discussed earlier backfired. Ostensibly, that provided additional motivation for the new leadership team to reassess the work of predecessors on this issue and committing, after consulting scientific evidence, to gradually phasing out animal testing.

Years of targeted advocacy by proponents of research innovation and animal-free science surely created permissive conditions for a tipping point to be reached in 2022. This did not go unnoticed by the new public health team and its decisive leadership that seized the opportunity to implement meaningful policy reforms. This administration provided the final blow that fractured path dependency, ostensibly for good.

### 11.3 The flawed “litmus test” for predicting drug response in humans is being off ramped

Drugs fail in human trials even though they have perfectly acceptable safety and efficacy profiles when tested on animals in preclinical stages (42). Yet over the years, animal testing has become the “litmus test” that dominates drug discovery. That is despite the poor predictive capacity of animal systems to forecast human response, and the dismal results produced by such arrangement.

According to *Clinical Development Success Rates* for 2011–2020, a key report from Biotechnology Innovation Organization (42), the largest association in the world representing the biotechnology industry clinical trials failure rate is 92% on average as mentioned earlier. Urology drugs have the highest failure rate (96%), followed by heart drugs (95%), cancer drugs (95%) and neurology drugs (94%). It is documented that failed oncology trials alone cost $50–$60 billion annually (59).

In fact, the exclusive reliance on animal testing has caused incalculable harm—the conventional animal-centric paradigm continues to falsely label toxic drugs as safe and leads to the premature abandonment of perfectly safe and effective drugs, simply because they fail animal testing. This paradigm also squanders the lives of millions of animals, overwhelmingly in vain.

Generally, therapeutic compounds fall into four categories: toxic in humans but not in animals, toxic in animals but not in humans, neither toxic in humans nor in animals, or toxic in both humans and animals. Efficacy patterns can be viewed from a similar vantage point. That is not factoring in the range of partial effects or the disparate outcomes among the many animal species, where even testing on different strains within the same species can show inconsistent results.

As illustrated in Table 4, regulators have historically allowed an experimental drug or vaccine to advance to the clinical stage only after favorable safety and efficacy outcomes in animals (namely *ti*, bright green). That said, and as designated by the blue area, a drug can be toxic and ineffective (TI), toxic and effective (Ti) or nontoxic and ineffective in animals (tI) but prototypically safe and effective in humans (ti). Vice versa, a drug can be perfectly safe and effective in animals (ti), but toxic or ineffective in humans, as documented in the stupefyingly (>90%) failure rate in clinical trials of investigational new drugs.

**Table 4:** A chart depicting the flaws in toxicity and efficacy assessments using animal-centric paradigms.

**Old Paradigm:** only positive outcomes in animals (*ti*) dictate the advancement of drugs or vaccines to clinical trials		Animals	Animals	Humans	Humans
		***T***	***t***	***T***	***t***
		**(Toxic)**	**(Not Toxic)**	**(Toxic)**	**(Not Toxic)**
Animals	***I***	*TI*	*tI*	*TI*	*tI*
	**(Ineffective)**				
Animals	***i***	*Ti*	*ti*	*Ti*	*ti*
	**(Effective)**				
Humans	***I***	*TI*	*tI*	*TI*	*tI*
	**(Ineffective)**				
Humans	***i***	*Ti*	*ti*	*Ti*	*ti*
	**(Effective)**				

To be read in conjunction with the corresponding text. Bright green (ti) represents favorable safety and efficacy outcomes in animals (or humans). Blue areas depict drugs that can be toxic and ineffective (TI), toxic and effective (Ti) or nontoxic and ineffective in animals (tI)—any of these can be safe and effective in humans (ti) but are discarded. A drug can also be perfectly safe and effective in animals (ti), but toxic or ineffective in humans, as documented in the high (>90%) failure rate in clinical trials of investigational new drugs due to safety or efficacy concerns.

Of note, experimental drugs are automatically abandoned after failing toxicity assessments in animals. It is hard to gauge the extent of the mislabeling of safe drugs in humans as toxic because of outcomes in animals. Nonetheless, examples from our daily routine shed light on the magnitude of this problem and the futility of the existing paradigms. Many household name drugs are indeed toxic in animals such as penicillin (fatal to guinea pigs), Tylenol (or acetaminophen, toxic in dogs and cats), and Aspirin (or acetylsalicylic acid, embryonically toxic in rats and rhesus monkeys).

Willow bark, which contains salicin (the active ingredient in aspirin) has been used for medicinal purposes dating back 4,000 years ago. In 1897, Bayer AG developed a synthetic form of salicin, naming it Aspirin. Acetaminophen (also known as paracetamol or Tylenol, a competitor to Aspirin and a household name too) was first synthesized in 1878 and first used to treat pain and fever in 1893. It is hard to believe that Aspirin or Tylenol would have most likely been rejected by regulators had they been synthesized after 1938 and subjected to “animal toxicity preclinical regulatory standards”. The hemorrhage of perfectly safe and effective drugs because of animal testing is maddening and irresponsible.

### 11.4 A final recap of breaking path dependency through the lens of the PESTEL framework

Admittedly, the speed at which change at U.S. agencies came about in April 2025 took everyone by surprise. Below, we attempt to examine the elements that enabled change through the lens of the PESTEL analysis framework. This strategic framework evaluates the external landscape by examining six factors: Political, Economic, Sociological, Technological, Ecological, and Legal.

***Political***. In the aftermath of the COVID-19 pandemic, questioning authority, including the public health system became a necessity. Legislators and citizens alike are now less comfortable with business as usual and the stifling bureaucracy that existed pre-pandemic. Of note, in the development of COVID-19 vaccine, animal testing requirement was essentially waived—“it is not required to demonstrate the efficacy of the SARS-CoV-2 vaccine candidate in animal challenge models prior to proceeding to FIH clinical trials”. Such landmark policy decision made at the March 2020 meeting of the International Coalition of Medicines Regulatory Authorities (ICMRA), which includes FDA, practically waived the requirement for animal testing before testing in humans. If this is acceptable when the stakes are high, it is fair to ask why can't that scenario be part of a sensible strategy to improve drug and vaccine development in other high priority conditions? It takes on average 12 years and $ 2 billion to develop a drug or vaccine. Finaly, a new administration in place focused on eliminating waste and reducing red tape was also a political factor driving forward change.

***Economic****.* The cost of failure alongside the sky-high expenses in drug development has reached a breaking point. It can no longer be sustained. This is now hurting every American. Regulatory acceptance of NAMs would provide drug sponsors more options for testing the safety and efficacy of drugs to improve clinical trial attrition rates, cut time to market in half, and substantially reduce R & D costs. A study by the biotech company Emulate concluded that routine use of the Emulate Liver-Chip (OOC) to identify liver toxicity risk in small-molecule drug development could generate approximately $3 billion per year by driving an increase in research and development productivity. Utilizing OOC to assess cardiovascular, neurological, immunological, and gastrointestinal toxicities could potentially generate around $24 billion per year in increased research and development productivity (56). A separate cost comparison analysis in 2023 by Moderna showed that a study using liver OOC) costs $325,000 compared to $5.25 million using NHPs, with experimental time reduced several folds using the OOC approach (53). The economic argument for pivoting away from animal testing is a convincing one, not only when it comes to reducing productivity loss but also for adopting more efficient systems and enhancing productivity gains (53, 56).

***Social***. Sustained pressure from advocates and concerned citizens around the world regarding the futile use of animals in research created a climate favorable for change. In 2018, the U.S. public was closely divided when it comes to the use of animals in scientific research. Some 47% favor the practice, while 52% oppose it, according to a Pew Research Center survey. But a 2024 survey from the PCRM finds most Americans favor ending animal research. Public opinion today is ostensibly favorable to eliminating invasive animal experimentation. Public opinion will likely be more favorable as the public become more aware of the scientific and ethical woes intrinsic to animal testing.

***Technological***. Advanced technologies cannot be ignored anymore (60). The many advantages of NAMs as alternatives to animal testing are discussed in Table 3. Federal agencies like the FDA and NIH, operating from an animal-centric bias since 1938 (as analyzed in this review) were easily co-opted to continue down the inefficient path by the powerful juggernaut of the animal industrial complex. They have now revised their strategic positioning with new, fastidious leadership and in the face of overwhelming scientific evidence supporting Alternatives.

***Ecological***. There is a growing realization that reducing and eliminating animal testing has an impact on our environment and healthy living. Animal testing depends on resource-intensive facilities, wildlife harvesting and global animal trades, all of which place direct and indirect pressures on ecosystems. Notably, seventy-five percent of infectious diseases and pandemics in humans are rooted in cross-species transmission—that is movement of pathogens from animals to humans (61). ***Legal***. U.S. legislation like FDAMA 2.0 (a U.S. law since 2022 and a critical juncture as outlined in this review) alongside draft bills (e.g., FDAMA 3.0, the CARGO Act) together with policy changes in Europe {e.g., EU resolution [P9_TA(2021)0387] for developing a wide action plan to end the use of animals in research and testing in September 2021} and endorsements from many professional organizations were all important contributors to change. In 2024, the American Bar Association, the largest voluntary professional association in the world which represents the legal profession in the United States, issued resolution 502 that “urges national governments, the U.S. Congress, and U.S. federal agencies to promote the development and use of methods that aim to replace, reduce, and refine the use of animal models in research and testing” and “urges national governments, the U.S. Congress, and U.S. federal agencies to remove barriers to, and create incentives for, the use of non-animal model research and testing methods in regulatory testing and federally sponsored research”.

## 12 Conclusions/final remarks

Most animal models have little resemblance to the human diseases they were created to study. And the exclusive reliance on animal testing translates today into irrecuperable delays in the development of medicines, missed opportunities due to misguided regulatory principles, and exorbitant costs ultimately passed onto consumers.

It appears though that implementing an accountable and transparent system of checks and balances built around the intentional use of innovative technologies in drug discovery is now underway at relevant U.S agencies. Path dependency related to animal testing is ostensibly in the process of being dismantled.

Admittedly, our interpretation of data and milestones listed in this work, while factual, benefits from the advantage of hindsight, which is twenty-twenty. Drug development is a complex, multifactorial process that is often coupled with financial, technical, administrative and sociological constraints, and where decisions are typically made under punishing deadlines. Besides, reaching a cure, where a disease is not expected to return, is often conflated with a “disease-free” status, a better characterization of the situation in most cases.

From that perspective, it can be argued that delaying the onset of a disease that presents at old age by a decade or two will markedly reduce its prevalence given life expectancy (75.8 years for men and 81.1 years for women). This thinking is impractical in pediatric illnesses, where a sick child can be contemplating lifelong treatments and a poor quality of life for decades. Moreover, rare diseases, where investments in drug development are generally scarce because of unassured financial returns represent a different set of challenges for drug developers. The small size of the patient population involved there translates into recruitment challenges for clinical trials, which influence statistical significance, which, in turn, adversely influence regulatory acceptance, at least in the existing paradigm.

The perspective is also different when examined through the lens of expenditures and costs. In the U.S., most of healthcare cost is attributed to chronic diseases. CDC states that “90% of the nation's $4.9 trillion in annual health care expenditures are for people with chronic and mental health conditions”. Addressing the burdens of high costs as a priority may reduce expenditure. But if efforts are not expanded equitably to other disease domains, this approach could be seen as unfair, even discriminatory. Health disparity and access to care is another layer of complication that drug developers and health administrators must consider, from the pricing of pharmaceutical drugs (which varies by country) to the logistics of practically delivering care. Finally, there is the vital element of personal responsibility since lifestyle choices correlate with health outcomes—the reliance on pharmaceutical drugs was never meant to be a permanent substitute for poor decisions an individual make in mitigating her or his health risk factors.

Reforming preclinical research in drug development will not fix all the above challenges but will be transformational in that it will demand critical thinking and proper disease modeling as the default approach to solving health predicaments.

The path forward is promising and wide open. But that still depends on the actions of many entities in the discovery space for the whole ecosystem must be open to change and reforms. Next to the evolving practices at U.S. health and regulatory agencies, better awareness of the unconscious biases that favors animal testing must occur in the communities of private funders, publishers, researchers, investors, educators, academic institutions and Wall Street. In some instances, change will entail forging a humane economy and a futility-free approach by confronting the conscious forms of biases exhibited by individuals and corporations benefiting from the *status quo.*

FDA commissioner Makary, NIH director Bhattacharya, EPA administrator Zeldin, Navy Secretary Phelan and leaders at CDC all have their work cut out for them. But if they persist with their plans to advance alternatives, they—alongside HHS Secretary Robert F. Kennedy Jr., and the current administration—may go down in history as the public health team that phased out futile animal testing from the regulatory process, brought relief to millions of Americans, and streamlined a massive healthcare industry, including a prescription drug marketplace, which in 2024, was valued at more than $1.5 trillion.

## Supplementary material

Supplementary Material 1 A letter to NIH director Dr. Jay Bhattacharya, concerning the unjustifiable funding for the National Primate Research Centers (NPRCs) and the urgency for bolstering Alternatives.

Supplementary Material 2 A bipartisan letter from lawmakers to then FDA commissioner robert califf inquiring about delays in updating the regulations related to FDAMA 2.0 and demanding the implementation of the new law.

Supplementary Material 3 A letter to NIH director Dr. Jay Bhattacharya, regarding the excluding dogs from NIH funding opportunities and bolstering Alternatives.

Supplementary Material 4 A report from the government accountability office (GAO) conveying to lawmakers the negative NIH response regarding legislation proposals to establish a national center for alternative research and bolstering animal-free methods.

## Glossary

FDA: food and drugs administration
NIH: national institutes of health
EPA: environmental protection agency
CDC: centers for disease control and prevention
EMA: European medicines agency
DON: department of the navy
VA: department of veterans affairs
CHE: center for a humane economy
AWA: animal wellness action
PeTA: people for the ethical treatment of animals
PCRM: physicians committee for responsible medicine
Federal FD&C Act: federal food, drug, and cosmetic act
FDAMA 2.0: FDA modernization act 2.0
NPRCs: national primate research centers
NCATS: national center for advancing translational sciences
ORIVA: office of research innovation, validation, and application
VQN: validation & qualification network
ICCVAM: interagency coordinating committee on the validation of alternative methods
ISTAND: innovative science and technology approaches for new drugs
GAO: government accountability office
OIRP: office of research infrastructure programs
OECD: the organization for economic co-operation and development
ISO: international organization for standardization
EURL ECVAM: European union reference laboratory for alternatives to animal testing
ICH: international council for harmonization of technical requirements for pharmaceuticals for human use
DILI: drug induced liver injury
KOMP: knockout mouse project
IMPC: international mouse phenotyping consortium
INDs: investigational new drugs
