# Supplementary material for: Path dependency and the rescuing of the biomedical research enterprise
Source: Front Med Technol. 2026 Jan 12;7:1683835. doi: 10.3389/fmedt.2025.1683835 (PMC12832638; doi:10.3389/fmedt.2025.1683835)
Supplement: Supplementary Material 4 — A report from the government accountability office (GAO) conveying to lawmakers the negative NIH response regarding legislation proposals to establish a national center for alternative research and bolstering animal-free methods. [file Supplementaryfile4.pdf]

RELEASED

RESTRICTED — Not to be released outside the General Accounting Office except on the basis of specific approval by the Office of Congressional Relations.

112169

13852

GAO

United States General Accounting Office
Washington, DC 20548

Human Resources Division

B-198011

MARCH 28, 1980

112169

The Honorable Norman F. Lent
House of Representatives

Dear Mr. Lent:

Subject: Proposed Legislation for Federal Funding to Develop Non-animal Alternatives for Research (HRD-80-69)

Your June 15, 1979, letter requested our comments on the feasibility and advisability of a legislative proposal to redirect 50 percent of Federal research funding for developing non-animal alternatives to research. In discussing this request with your office, we pointed out that we had received a related request from Congressman Charles Pashayan, Jr., to review several issues regarding non-animal alternatives.

As your office agreed, this letter presents our views on the legislative proposal and the information we obtained in responding to Congressman Pashayan. We discussed the use of alternatives with the National Institutes of Health (NIH) officials and representatives of various private organizations concerned with biomedical research and animal welfare. We also reviewed literature related to this subject, recommended to us by several persons we interviewed.

BACKGROUND

The term "alternatives," when applied to the use of animals in biomedical research, is generally recognized as covering one or more of the following possibilities:

--Not using any laboratory animals.

--Reducing the number of animals required.

--Refining existing methods to minimize the amount of animal suffering and stress.

(103962)

509356

HEW AGC000[illegible]
NIH AGC00028

AGC00368 (APHIS)

Animal welfare rights organizations have taken the position that several alternatives are available in lieu of using animals and that the research community is not effectively using alternatives whenever possible.

NIH acknowledges that alternatives are available and believes that they have been used in research as they have become available. NIH contents that there is an incentive to adopt such techniques, to the extent that alternatives can provide more reliable information and reduce the cost of experiments.

The issue, it appears, is not whether there are alternatives to the use of animals for research, but how quickly and under what circumstances they should be used. Researchers have generally taken the position that there is little opportunity for complete replacement of laboratory animals. Although animals can be eliminated from certain phases of experiments and tests by using alternative methods, researchers believe that using some animals will still be necessary. For example, tissue cultures are a frequently cited alternative to animals; however, animals are required to provide the tissue. The greatest potential, therefore, is in finding the means for reducing the number of animals used.

LEGISLATIVE PROPOSALS

Several bills were introduced in the 96th Congress pertaining to alternatives to the use of live animals in research and testing. The Research Modernization Act (H.R. 4805) most directly relates to the issues discussed in your letter. This legislation calls for:

--Establishing a National Center for Alternative Research.

--Developing and coordinating alternative methods of research and testing not involving the use of live animals.

--Designing training programs in the use of alternative methods.

--Eliminating duplication of research and testing of live animals.

--Disseminating information on alternative methods.

Also, it provides for redirecting between 30 and 50 percent of the total Federal research funds involving the use of live animals to develop alternative methods.

H.R. 4805 was introduced in response to a proposal by an animal welfare rights organization that had expressed concern that the research community was not effectively using non-animal alternatives for research and testing whenever possible. However, action has not been taken on this or similar legislation.

NIH, in commenting on H.R. 4805, stated that while the intent to promote animal welfare was commendable, legislation of the scope and nature proposed was both unnecessary and unworkable. NIH noted that the bill would prohibit the use of funds for animal testing once an alternative is identified. It argued that the results from research and tests involving the use of alternatives are often validated through the use of live animals--an essential step in determining possible effects on human health and safety. NIH also said that duplicative research and testing, in which the results of one investigator are confirmed or disproved by another, is an important part of the scientific process.

NIH further pointed out that requiring extensive reprograming of funds from live animal research to alternatives would severely limit support for a large number of important research programs and hamper scientific progress in many areas of biomedical research. It added that the amount of funds proposed for developing alternative research methods could not be wisely expended when basic knowledge and technology are lacking.

CURRENT FEDERAL GUIDANCE ON USING ANIMALS IN RESEARCH

There is currently no legislation or regulation that pertains directly to alternatives for using laboratory animals in research. The primary legislation concerning the use of laboratory animals in research is covered under the Animal Welfare Act. Its object, with respect to laboratory animals,

is to ensure that animals intended for use in research facilities are provided humane care and treatment. The act does not, however, address the need for using animals in research or promoting alternative methods.

The Department of Agriculture's Animal and Plant Health Inspection Service (APHIS) is responsible for administering and enforcing the Animal Welfare Act. The act covers a broad range of animal welfare issues involving specific types of animals, such as hamsters, guinea pigs, rabbits, dogs, cats, primates, and most other warm-blooded animals. Exempted from the act are rats, mice, birds, marine animals, farm animals, and all cold-blooded animals. Since rats and mice comprise the largest number of laboratory animals, many of the animals used for research purposes are not covered by the act. An APHIS official stated that consideration is being given to including rats and mice under the act, since such animals are now included under the Food and Drug Administration's regulations on laboratory management practices.

APHIS conducts recurring unannounced inspections of research facilities to determine compliance with its regulations and standards. During fiscal year 1978 APHIS conducted about 4,000 such inspections.

APHIS requires each research facility to submit an annual report showing the number and types of animals used. Also, it identifies the number of animals that experienced pain or distress without the use of pain-relieving drugs where the use of drugs would interfere with the research or testing purpose. According to the fiscal year 1978 report, about 1.9 million animals were used in experiments during that year, of which about 150,000 (or 8 percent), were reported experiencing pain. An APHIS official acknowledged that such information is not routinely validated, but that some means of validation is being considered.

It should be noted that the statistics cited in the APHIS report on animal usage pertain only to animals covered by the Animal Welfare Act. Little information is available on the total number of animals used in biomedical research nationwide.

The primary source of such information is the National Academy of Sciences's Institute for Laboratory Animal Resources. The Institute conducts occasional surveys of animal facilities and resources. Its most recent published survey, for the years 1967 and 1968, showed that over 33 million warm-blooded animals were used annually for research purposes. In 1979 the Institute conducted another survey; however, the results were not available as of February 1980. Until 1971 the Institute published annual statistics on the number of animals used in research. However, it discontinued such reports when APHIS began publishing data on animal usage as required by the Animal Welfare Act. The 1971 report showed that researchers used at least 45 million animals annually. Except for the APHIS reports discussed above, this is the latest known information available from government-sponsored studies.

The most recent estimate that we noted was contained in a report 1/ of the Institute for the Study of Animal Problems, a Division of the Humane Society of the United States. It stated that the current demand for laboratory animals is estimated at about 100 million, including about 70 million rats and mice.

NIH has issued policy statements prescribing policies and responsibilities for the humane care and use of animals in NIH grant-supported activities. The policy provides that before receiving a grant in which animals are used, an institution must submit to NIH an assurance that it will comply with NIH's "Guide for the Care and Use of Laboratory Animals" and its statement of Principles for the Use of Animals. The Guide, first published in 1963, was prepared for NIH by the Institute of Laboratory Animal Resources.

Its purpose is to assist scientific institutions in the use and care of laboratory animals. The Guide provides extensive information regarding housing, sanitation, husbandry, and veterinary care for laboratory animals, but does not say when animals may be used or when alternative methods should be considered.

1/"Alternatives to Laboratory Animals" by Andrew N. Rowan.

In November 1978, NIH issued a revised policy statement on the care and use of animals. The policy incorporates NIH's Principles for the Use of Animals. As with the Guide, the policy is basically concerned with humane treatment of animals used in research. The 1978 revision, however, contains provisions relating to alternative methods of research. Any grant application involving the use of animals must provide information on the rationale for using the animals. NIH officials believe that the inclusion of such information will allow grant reviewers to assess whether the use of animals is justified. In addition, the statement of NIH Principles provides that statistical analysis, mathematical models, or *in vitro* 1/ biological systems should be used, when appropriate, to complement animal experiments and to reduce the number of animals used.

NIH RESOURCES DEVOTED TO ALTERNATIVES

NIH does not have complete information on the amount of research funds for non-animal research or for developing other alternatives. Often such research efforts would be included as subobjectives in research studies by various institutes which focus on the study of a particular disease. During such research, alternative methodologies may be used in conducting all or part of the research.

According to NIH officials, the principal identifiable source of funding such alternatives would be its Division of Research Resources. The Division supports the development and application of a wide range of methods and technology to aid scientists in conducting biomedical research. The Biotechnology Resources Program is the focal point within the Divison for developing research and testing methods and technology which do not require the use of animals. It is the only program at NIH directed exclusively to providing researchers access to improved methods and sophisticated technology. The funds for developing such methods and technology are about $14 million annually. Examples include the development of mass spectrometry, electron microscopy, and sophisticated computer systems. According to Division officials, successful development of new methods and technology should reduce the number of experiments needed to

---

1/Refers to experiments using tissue or cell cultures rather than living animals.

confirm and substantiate new research ideas, thereby reducing the number of animals required.

During the latter part of 1979, NIH conducted a study to determine the extent to which NIH-sponsored research projects used or developed alternatives to the use of laboratory animals. In that study, alternatives were defined as systems or procedures that could reduce the (1) number of animals used or (2) amount of pain and suffering of animals involved in meeting a particular scientific objective. The most common examples of alternatives were considered to be in vitro techniques, mathematical models, and clinical and epidemiological investigations.

This information was obtained principally by a computer search of selected NIH research projects. The descriptors used in the search included more than 30 standard terms, such as tissue culture and computer simulation, designed to identify projects that (1) used alternatives or (2) relied heavily on laboratory animals. Projects were included in the final tabulation only if they placed primary emphasis on the selected descriptors and involved the use of alternatives.

The study identified nearly 1,000 projects funded in fiscal year 1978 costing about $192 million (about 7 percent of the total NIH research budget). The projects were divided among four general categories: toxicity testing and screening, preparation and standardization of drugs, basic experimental research, and biomathematics.

NIH PLANS FOR RESPONDING TO CONCERNS OVER NEED FOR PROMOTING ALTERNATIVES

As a result of its study, NIH believes that several issues need to be addressed before considering whether or to what extent additional funds should be spent to further promote the use of alternatives. NIH is developing plans for a conference later this year to address advantages and limitations of alternatives to the use of laboratory animals. The major objectives of this conference are to:

- --Assess the technical status of research in the area of production and standardization of biologicals, the potential for reducing the use of laboratory animals, and the adequacy of current support.

--Evaluate the advantages and limitations of *in vitro* methods that would serve as alternatives to *in vivo* 1/ tests, the potentials for strengthening the *in vitro* methods, and the level of support needed.

--Evaluate the potential for strengthening *in vitro* and *in vivo* testing methods by broadening the science base in toxicology, and the adequacy of current research support.

--Determine whether opportunities exist to conduct epidemiological and clinical studies designed to develop a better understanding of the relevance of *in vitro* and animal bioassay tests to human health.

--Consider whether opportunities exist for mathematical applications that may lead to more efficient use of research resources.

According to NIH officials, the conference is a necessary first step in determining the extent to which (1) further research is needed on developing alternatives to the use of animals, and (2) greater use of available alternative methods is warranted. Where further research is considered necessary, NIH could issue requests for applications to identify potential sources for developing alternatives.

NIH officials believe that adequate sources of information on alternative research methods are available to researchers. Three of the principal sources of information cited were the National Library of Medicine, the Smithsonian Science Information Exchange, and publication of research studies in scientific journals. The officials stated that publication of research results is a primary objective in all research studies and is considered an effective means of informing others of NIH-funded research results. Thus, NIH does not believe it is necessary to have additional dissemination efforts. However, the officials noted that the planned conference would provide an opportunity to assess whether any further efforts should be considered.

1/Refers to experiments using live animals.

CONCLUSIONS

There is much concern about the use of animals in research. We believe that more information is needed on the advantages and limitations of alternative methods and the extent of inappropriate animal experimentation before deciding whether legislation, such as H.R. 4805, is needed.

The extensive reprograming of research funds proposed by H.R. 4805 should be delayed pending further study. The planned NIH conference could provide beneficial information on the use of alternatives. NIH should then be in a better position to inform the Congress of the extent to which additional funds are needed and could be used effectively for developing further research alternatives and in seeking ways to achieve greater use of available alternative methods.

Additional funding would not necessarily require new legislation. The Congress has on several occasions identified areas of special concern which it believed warranted increased funding. The Appropriations Committees have requested NIH to submit reports on the status of, and plans for, various programs and, in several cases, requested NIH to place additional emphasis on certain research areas. Nutrition research is a recent example of an issue that cuts across all NIH program areas for which the Congress believes additional emphasis is needed. No specific legislation was enacted to authorize funding for nutrition research. However, the Congress increased NIH appropriations under its existing authorization and directed NIH to use a portion of the increased funding for additional nutrition research.

Similarly, the Congress could request a status report from NIH on its efforts in developing and using alternatives. If the Congress believes additional funding is warranted, it could direct NIH, under existing legislation, to place greater emphasis on such research. By monitoring NIH's efforts and assessing the impact of additional funding, the Congress could determine whether additional legislation is needed.

\- - - -

As arranged with your office, unless you publicly announce its contents earlier, we plan no further distribution of this report until 30 days from the date of the report. At that time we will send copies to interested parties and make copies available to others upon request.

Sincerely yours,

Gregory J. Ahart
Director
