# Supplementary material for: Path dependency and the rescuing of the biomedical research enterprise
Source: Front Med Technol. 2026 Jan 12;7:1683835. doi: 10.3389/fmedt.2025.1683835 (PMC12832638; doi:10.3389/fmedt.2025.1683835)
Supplement: Supplementary Material 1 — A letter to NIH director Dr. Jay Bhattacharya, concerning the unjustifiable funding for the National Primate Research Centers (NPRCs) and the urgency for bolstering Alternatives. [file Supplementaryfile1.pdf]

animal wellness action

CENTER FOR A HUMANE ECONOMY

May 13, 2025

The Honorable Jayanta Bhattacharya
Director, National Institutes of Health (NIH)
9000 Rockville Pike
Bethesda, Maryland 20891

Dear Dr. Bhattacharya,

On behalf of the consortium of animal welfare groups I lead, we celebrated your landmark announcement seeking to "reimagine the way research is conducted" and to move toward replacing animal models at NIH with 21st-century human-relevant strategies. You followed that game-changing reset of research in America with a logical step to close the last remaining in-house beagle laboratory on the NIH campus.

These foresighted initiatives undoubtedly give hope to your many stakeholders, including millions of patients. For far too long, archaic policies and unreliable practices held back NIH's progress and eroded the public trust in its capacity to deliver game-changing health outcomes for patients in crisis. You are restoring trust by "ushering in a new era of innovation."

Bolstering the reliance on advanced technologies, including New Approach Methodologies (NAMs), to substitute for unreliable animal-centric research paradigms[1] is not only a welcome new practice at health agencies but also a requisite for pathbreaking discoveries in public health. Your vision statement came just two weeks after FDA commissioner Makary announced a roadmap to phase out animal testing for monoclonal antibodies (mAbs) and other classes of drugs at FDA. EPA Administrator Zeldin also announced the reinstatement of a policy from the first Trump administration to phase out animal testing at the agency, an effort that was gratuitously paused and tabled by the Biden administration.

We are grateful to the new public health leadership team, including HHS Secretary Kennedy, for not wasting a moment to provide a critical reboot of strategies for screening of drugs and chemicals and conducting basic research in our nation's most important scientific settings.

Here, we note that the expansive NIH-funded network of resources and centers, including the National Regional Primate Centers (NPRCs), is part of the ossified infrastructure built around the use of unreliable animal models. After 60 years of squandering hundreds of billions of federal funds, it's time to redirect funding from such a large primate network to the qualification and development of human-relevant NAMs.

- The seven NPRCs alone received approximately $100 million in 2023 for base grants and other support, despite acknowledgement that primate use is unsustainable and unethical.[2]
- FDA prioritizing phasing out testing on mAbs, which are mainly tested on non-human primates (NHPs), was not arbitrary. Decades of similar testing have shown the unreliability of this approach and the need to eliminate the dependency on NHPs.
- Antibody testing typically requires 150 primates (avg. $50,000 each), so a cohort will cost $7.5 million. A cost comparison analysis from Moderna in 2023 showed that an experiment using

[1] Pound, Pandora, et al, (2014) Is animal research sufficiently evidence based to be a cornerstone of biomedical research?, *BMJ* 2014; 348 doi: https://doi.org/10.1136/bmj.g3387.

[2] Bailey J, Taylor K. Non-human primates in neuroscience research: the case against its scientific necessity. Altern Lab Anim. 2016;44(1):43-69. doi:10.1177/026119291604400101

animal-free organ-chips cost $325,000 compared to $5.25 million using NHPs, with experimental time reduced several fold when using organ-chips.

- It would be an uneven and wasteful policy to implement the FDA roadmap within 3-5 years without stalling funding for NPRCs given the decades-long lifespans of primates.

Continued Use of Monkeys is Unsound Science

The limited predictive value of nonhuman primate models for human biology, combined with the availability of more precise human-based methods, nullifies the justification for using NHPs in medical testing and research. The continued reliance on NHPs will slow progress toward effective cures and therapies.The biological and genetic differences between NHPs and humans limit the reliability of data extrapolated from primates to humans.

There are also concerns about public health consequences for importing NHPs and for breeding them domestically. The medical establishment has considered the shortage of these animals, caused by reduced imports from China during and after Covid-19, a "crisis," and with this contrived and false urgency has come criminal conduct, including the smuggling and import of endangered NHPs.

Investigations by veterinary and health authorities found that inadequate safety measures at Vietnamese monkey breeding and export facilities allowed tuberculosis bacteria to spread there and to labs around the world that purchased infected animals. In 2019 a long-tailed macaque scheduled for stem-cell research at the Oregon Health and Science University tested positive for TB, rendering the entire shipment unusable.

The New England Regional Primate Center was shuttered more than a decade ago, and Oregon Governor Tina Kotek recently and publicly advocated for the closure of the Oregon National Primate Research Center (ONPRC), operated by Oregon Health & Science University (OHSU). In late March 2025, her office confirmed that she urged OHSU leadership to complete their current research obligations and move toward shutting down the center in a humane and responsible manner.

I urge you to move with all deliberate speed to wean federal funding from the network of primate centers, signaling that these institutions to wind down this form of their work. We also urge you to end intramural primate research, replicating your decision-making with beagles. Additional investments of billions of federal tax dollars in the years ahead for the NRPCs will just produce more primates for drug screening and other types of testing and experimentation that the Trump leadership team has rightly recognized as wasteful and inefficient.

The use of primates in testing is obsolete now, and their use will look even more irresponsible in five or ten years or beyond. We must stop putting primates, with lifespans of up to 40 years, into the system.

Animal Wellness Action and the Center for a Humane Economy launched the FDA Modernization Act campaign in 2021 to eliminate the animal testing mandate and worked with Republicans and Democrats to pass the FDA Modernization Act 2.0. We continue to push for the implementation of the new law through the bipartisan FDA Modernization Act 3.0 (S. 355, H.R. 2821).

Our request to wind down the work of the primate centers and related resources is consistent with your work, Dr. Makary's, and Congress's unanimous approval of the FDA Modernization Act. The NIH and other public-health agencies cannot logically stand by their clear-eyed vision for 21st-century drug screening and basic research in America and then allow the old, inefficient machinery of animal models to remain in place.

Your stirring and foresighted words compel a careful disassembly of the elements of a long-broken experimental drug development process that has driven up drug prices, delayed delivery of drugs to market, limited the range of experimental drug protocols, and victimized countless millions of animals.

Thank you again for your very encouraging and inspirational work in this space.

Sincerely yours,

Wayne Pacelle
President
Animal Wellness Action

Zaher Nahle, PhD, MPA
Senior Scientific Advisor
Center for a Humane Economy
