# Supplementary material for: Path dependency and the rescuing of the biomedical research enterprise
Source: Front Med Technol. 2026 Jan 12;7:1683835. doi: 10.3389/fmedt.2025.1683835 (PMC12832638; doi:10.3389/fmedt.2025.1683835)
Supplement: Supplementary Material 2 — A bipartisan letter from lawmakers to then FDA commissioner robert califf inquiring about delays in updating the regulations related to FDAMA 2.0 and demanding the implementation of the new law. [file Supplementaryfile2.pdf]

16 November 2023

Robert M. Califf, M.D.
Commissioner
U.S. Food and Drug Administration
10903 New Hampshire Avenue
Silver Spring, MD 20993

Dear Dr. Califf:

We write to you about the investigational new drug development work of the FDA in the wake of the enactment of the FDA Modernization Act 2.0.

That legislation, which Congress passed and the President signed into law at the end of 2022 with uncommon unanimity, removes FDA's mandate for animal testing of all new drug candidates and allows an applicant for new drug market approval to use methods other than animal testing to establish a drug's safety and effectiveness. During one of your appearances before the U.S. Senate Committee on Health, Education, Labor, and Pensions, you spoke favorably about the shift toward human-based biology, noting that alternative methods may include cell-based assays, organ chips and micro-physiological systems, computer modeling, bioprinting, and a growing variety of other New Approach Methodologies (NAMs).

As you are aware, agency regulations are promulgated in accordance and conformity with Congress's statutory language and intent. Yet in the aftermath of the enactment of the FDA Modernization Act 2.0, the FDA's regulations related to animal testing no longer fully conform with applicable law. For example, federal regulations governing submission of Investigational New Drug (IND) applications require that amendments to an IND "should be supported by additional information, including the results of animal toxicology studies or other human studies as appropriate."[1] Further, regulations governing the IND Investigator's Brochure call for a summary of "pharmacological and toxicological effects of the drug in animals and, to the extent known, in humans."[2] These and other regulatory provisions no longer reflect the full scope of the governing statute and should therefore be updated as expeditiously as possible.

The above examples are just a sampling of a larger set of inconsistencies between the amended statute and FDA regulations. We therefore write to ask what specific steps the FDA is taking to update its animal testing regulations, and what its timeline is for implementation of a revised regulatory framework. Please respond with this information within 30 days of the date of this letter.

---

[1] *See* 21 C.F.R. § 312.22(c).

[2] *See* 21 C.F.R. 312.23(a)(5)(ii).

Thank you for your attention to this important matter.

Sincerely,

Rand Paul, M.D.
United States Senator

Cory Booker
United States Senator

Mike Braun
United States Senator

Angus King
United States Senator

Roger Marshall, M.D.
United States Senator

Tim Kaine
United States Senator

John Kennedy
United States Senator

Ben Ray Luján
United States Senator

Eric S. Schmitt
United States Senator
