# Supplementary material for: Path dependency and the rescuing of the biomedical research enterprise
Source: Front Med Technol. 2026 Jan 12;7:1683835. doi: 10.3389/fmedt.2025.1683835 (PMC12832638; doi:10.3389/fmedt.2025.1683835)
Supplement: Supplementary Material 3 — A letter to NIH director Dr. Jay Bhattacharya, regarding the excluding dogs from NIH funding opportunities and bolstering Alternatives. [file Supplementaryfile3.pdf]

CENTER FOR A HUMANE ECONOMY

animal wellness action

May 27, 2025

The Honorable Jayanta Bhattacharya
Director, National Institutes of Health (NIH)
9000 Rockville Pike
Bethesda, Maryland 20891

Dear Dr. Bhattacharya,

I wrote to you recently, on behalf of Animal Wellness Action and the Center for a Humane Economy, urging a wind down the work of the federally funded network of Primate Centers. Today, I write on the related topic of beagles bred and housed for use in invasive experiments and ask you to modify your grant-making process to disfavor the use of beagles for recipients of federal dollars.

You've already been very active on this subject and closed the last intramural beagle research facility on the NIH campus. With our great appreciation for that humane action, we hope that you'll build on it and stipulate exclusion criteria in all new Funding Opportunity Announcements (FOA), disallowing the use of beagles as a model system. Like you, we are increasingly concerned by the continued use of dogs in NIH-funded research as a matter of ethical concern and scientific reliability.

Dogs, especially beagles, are often selected for research because of their docile temperament and manageable size. These characteristics are logistical conveniences—not indicators of scientific appropriateness.

Published data demonstrate that dogs, including beagles, are poor predictors of drug response in humans due to fundamental differences in metabolic, genetic, physiological, and pharmacological difference between the two species. In fields such as heart disease, kidney disease, ALS, cancer and metabolic syndromes, canine models have failed to mimic disease progression and treatment response in people.

Dogs are animals known for their intelligence, loyalty, and deep emotional capacities, and there is a deep connection among Americans to their well-being in all settings. The use of dogs in invasive experiments not only causes considerable suffering but is increasingly out of step with public sentiment and the direction of modern science that you have embraced.

We respectfully call on the NIH to devise an actionable plan to phase out dogs from taxpayer-funded grants. This can begin by starting with beagles as a first step, excluding their procurement in all new Funding Opportunity Announcement (FOA) notices. Of note, in November 2015, NIH terminated the use of chimpanzees as a model system and phased out all active biomedical research protocols using these animals. The Institute of Medicine determined that chimpanzees were no longer needed for life-saving research and testing. Based on your comments and similar statements from Dr. Makary about the problems with animal models, along with the benefits from the use of human-relevant methods, the same conclusion can be drawn on the use of dogs in testing and research.

We further urge the agency to increase investment in advanced, non-animal research models that align with your stated commitment to 21st-century science. Leading the way in this policy shift would reinforce NIH's commitment to both scientific excellence and ethical responsibility.

We cannot sufficiently convey our appreciation for your important statements on the future direction of human-relevant science. We are immensely grateful for your dedication to public health and look forward to seeing continued progress in the direction of humane and cutting-edge research.

We included in the appendix a few accounts of demonstrably inhumane treatment of beagles bred for research in the United States for the purpose of use in testing and research.

Sincerely,

Wayne Pacelle

President

Animal Wellness Action

Zaher Nahle, PhD, MPA

Senior Scientific Advisor

Center for a Humane Economy

Appendix

Just Two Breeding Facilities Supplying Beagles to Laboratories Operate

Neglect and cruelty are par for the course

Ridglan Farms, Blue Mounds, Wisc.

Inhumane Housing Conditions

- Dogs confined in small, stacked wire cages within windowless sheds, lacking outdoor access or social interaction.
- Cages with flooring that caused foot injuries, including sores and blisters.
- Unsanitary conditions, with excrement accumulation and inadequate ventilation.

Unlicensed Surgical Procedures

- Surgeries such as debarking and "cherry eye" removal performed by unqualified staff without anesthesia or pain relief.
- Lack of veterinary supervision during these procedures.

Neglect and Psychological Distress

- Untreated injuries and infections among the dogs.
- Signs of psychological distress, including repetitive behaviors and self-harm.

Regulatory Non-Compliance

- Repeated violations documented by the Wisconsin Department of Agriculture, Trade and Consumer Protection (DATCP), including inadequate sanitation, ventilation, and veterinary care.
- Failure to address issues despite prior inspections and warnings.

Marshall BioResources, Wayne County, N.Y.

Inhumane Training Practices: In October 2024, PETA released footage from MBR's New York facility, showing beagles restrained with tight-fitting inhalation masks. These masks are used in chemical testing, and the dogs appeared distressed, struggling to remove them. MBR claims that the dogs were "trained" to wear the masks, labeling it as "learned helplessness".

Unsanitary Living Conditions: A whistleblower report in June 2024 highlighted that beagles and ferrets were confined in filthy cages with dirty food and water containers. The report also alleged that puppies born with physical "imperfections," such as different-colored eyes, were killed at birth.

Regulatory Actions: Since 2007, the USDA has cited MBR for countless violations of the Animal Welfare Act, including inadequate veterinary care and poor living conditions.
